# Characterisation of the symbionts in the Mediterranean fruit fly gut

**DOI:** 10.1099/mgen.0.000801

**Published:** 2022-04-21

**Authors:** Mike Darrington, Philip T. Leftwich, Neil A. Holmes, Lucy A. Friend, Naomi V. E. Clarke, Sarah F. Worsley, John T. Margaritopolous, Saskia A. Hogenhout, Matthew I. Hutchings, Tracey Chapman

**Affiliations:** ^1^​ School of Biological Sciences, University of East Anglia, Norwich Research Park, Norwich, NR4 7TJ, UK; ^2^​ Department of Molecular Microbiology, John Innes Centre, Norwich Research Park, Norwich, NR4 7UH, UK; ^3^​ Department of Plant Protection, Institute of Industrial and Fodder Crops, Hellenic Agricultural Organization–DEMETER, Volos, Greece; ^4^​ Department of Crop Genetics, John Innes Centre, Norwich Research Park, NR4 7UH, Norwich, UK

**Keywords:** *Klebsiella*, symbiont, paratransgenesis, 16S rRNA sequencing

## Abstract

Symbioses between bacteria and their insect hosts can range from loose associations through to obligate interdependence. While fundamental evolutionary insights have been gained from the in-depth study of obligate mutualisms, there is increasing interest in the evolutionary potential of flexible symbiotic associations between hosts and their gut microbiomes. Understanding relationships between microbes and hosts also offers the potential for exploitation for insect control. Here, we investigate the gut microbiome of a global agricultural pest, the Mediterranean fruit fly (*Ceratitis capitata*). We used 16S rRNA profiling to compare the gut microbiomes of laboratory and wild strains raised on different diets and from flies collected from various natural plant hosts. The results showed that medfly guts harbour a simple microbiome that is primarily determined by the larval diet. However, regardless of the laboratory diet or natural plant host on which flies were raised, *Klebsiella* spp. dominated medfly microbiomes and were resistant to removal by antibiotic treatment. We sequenced the genome of the dominant putative *Klebsiella* spp. (‘Medkleb’) isolated from the gut of the Toliman wild-type strain. Genome-wide ANI analysis placed Medkleb within the *K. oxytoca / michiganensis* group. Species level taxonomy for Medkleb was resolved using a mutli-locus phylogenetic approach - and molecular, sequence and phenotypic analyses all supported its identity as *K. michiganensis *. Medkleb has a genome size (5825435 bp) which is 1.6 standard deviations smaller than the mean genome size of free-living *Klebsiella* spp. Medkleb also lacks some genes involved in environmental sensing. Moreover, the Medkleb genome contains at least two recently acquired unique genomic islands as well as genes that encode pectinolytic enzymes capable of degrading plant cell walls. This may be advantageous given that the medfly diet includes unripe fruits containing high proportions of pectin. The results suggest that the medfly harbours a commensal gut bacterium that may have developed a mutualistic association with its host and provide nutritional benefits.

## Data Summary

**Impact Statement**

This research investigates the extent and nature of the relationship between hosts (here the Mediterranean fruit fly, aka medfly) and their gut microbiomes. It builds upon previous work in keys ways: (i) to profile both culturable and non-culturable gut microbiomes of wild- and laboratory-derived adults from a range of different larval diets, and (ii) to conduct the first long-read genome sequencing and analysis of the genome of the dominant, putative *Klebsiella* spp. symbiont. The results suggest that the medfly consistently harbours a commensal gut bacterium with which it may have developed a mutualistic association. The results are important in showing the significance of the evolutionary potential of flexible symbiotic associations between hosts and their gut microbiomes. Understanding this relationship also offers the potential for exploitation for insect control of this global agricultural pest.

All sequencing data has been submitted to the NCBI Sequence Read Archive and is available under BioProject ID PRJNA638617. Scripts and metadata to reproduce all figures can be accessed via the GitHub repository https://github.com/Philip-Leftwich/Characterisation-of-the-symbionts-in-the-Mediterranean-fruitfly-gut. The authors confirm all supporting data, code and protocols have been provided within the article or through supplementary data files.

## Introduction

All eukaryotic organisms host bacteria [1] and some of the best-studied associations are those that occur between insects and bacteria [2–4]. The majority of microbe-insect interactions are temporary associations. However, some bacteria and insects retain persistent associations over evolutionary time and have evolved co-dependence [4]. In the most extreme examples, these associations have persisted for millions of years and represent intimate co-evolutionary relationships [4, 5]. For example, heritable symbioses are defined by the direct passage of bacteria from insects to progeny, usually via maternal transmission [4]. These symbioses can be facultative or obligate, from both the host and microbe’s perspectives [4]. Over evolutionary time, facultative symbionts that can only live within their hosts may lose genes that facilitate life in varied environments [3]. A transition to evolutionary interdependence with hosts can often be identified in bacterial symbionts by reduced genome size, deletion of loci and reduced GC content [6–10]. Novel loci can also be acquired via lateral gene transfer [11, 12]. Mutualistic benefits provided to insects by bacteria include synthesis of nutrients [13–15], carotenoids [16], and antipredation molecules [14, 17].

In addition to classic mutualisms, there is an increasing body of research into the identities and potential roles of diverse symbionts found across many different taxa [18–24]. This includes the study of microbes that are apparently only loosely associated with their hosts, such as the gut symbionts of the Dipteran Tephritids (or ‘true’) fruitflies that are the focus of this study. The potential utility of symbionts to either provide new routes for pest control or to improve existing technologies [25–27] has led to additional interest in investigating the symbiotic gut microbial communities of key global pests, such as the medfly [28–30]; studied here.

Dipteran gut microbiomes are often relatively stable and simple [31–33] and can be strongly influenced by host diet [31, 34]. For example, the gut microbiome of the fruit fly *Drosophila melanogaster* can comprise a high abundance of Lactobacillales spp. when reared on complex polysaccharide diets but is dominated by Acetobacteraceae or by γ-Proteobacteria on high sugar diets [35] While the microbiota of wild flies is more diverse, these same bacteria continue to dominate in natural populations [35]. This suggests that adult hosts of *D. melanogaster* may actively regulate their gut microbiomes [36–38] and potentially gain benefits from doing so [33]. For example, core members of the *D. melanogaster* gut microbiome metabolise lactic acid and acetic acid, which may benefit larvae feeding on rotten fruit. In contrast, the larvae of Tephritid fruitflies hatch in unripe fruit and the culturable species within their gut microbiomes are reported to contain pectinolytic bacteria, which could assist the host in breaking down plant cell walls [39–41].

Investigations of culturable gut bacteria in the Tephritid *Ceratitis capitata* (Mediterranean fruit fly, or medfly) on which we focus in this study, have been performed predominantly using amplified rRNA restriction analysis (ARDRA). These show that *Klebsiella* spp. of bacteria comprise at least 20–30 % of the larval, pupal and adult medfly gut microbiomes [29, 42, 43]. This has led to the hypothesis that one of the main *Klebsiella* species identified, *K. oxytoca,* might provide benefits in terms of larval nutrition via its reported ability to break down fruit sugar (pectin), or to fix nitrogen [39]. A 16S rRNA analysis has also been used to show that irradiation, which is often used to produce infertile males as part of Sterile Insect Technique (SIT) control programmes, diminishes the relative contribution of *Klebsiella* spp. to the medfly microbiome [29]. Subsequent reintroduction of *K. oxytoca* to irradiated flies caused a significant reduction in male mating latency (i.e. more rapid remating) in comparison to males fed a control sterile diet without bacteria [29]. This suggested a potential benefit to the host’s fitness associated with the presence of *K. oxytoca* in the gut.

Although the culturable species within medfly microbiomes have been described, many details remain unclear. For example, we do not yet know the contribution of non-culturable species, whether microbiomes are stable, whether *Klebsiella* spp. have the capability to confer a direct fitness benefit to the medfly, and the extent to which *Klebsiella* is heritable. Behar *et al*. [39] suggest that a gut symbiont identified as *K. oxytoca* is heritable and can be transmitted during oviposition. However, in that study, *K. oxytoca* was detected in only one of four replicates of pooled eggs [39] and hence the transmission of *K. oxytoca* during oviposition was not reliably demonstrated . Recovery of GFP-labelled *Klebsiella* bacteria in the guts of offspring of mothers into which those bacteria were experimentally introduced, provides evidence of vertical transmission [44], though the relative extent of such transfer is not yet clear. In terms of fitness benefits, Gavriel *et al*. [30] experimentally depleted the microbiome of male medflies by using irradiation and fed males with a diet either containing *K. oxytoca* or a sterile diet. *K. oxytoca-*fed males outcompeted sterile-diet males for matings, and females mated to *K. oxytoca-*fed males were less inclined to re-mate. These data suggest that *Klebsiella* spp. have the potential to confer benefits to hosts [29, 30, 43] though it is not yet clear whether this occurs in the natural context.

Here, we extended previous studies of medfly gut microbiomes in several key ways. First, we compared the culturable and non-culturable gut microbiomes of wild-collected adult medflies from a range of different wild hosts, with those of a wild-type laboratory strain reared on a range of different larval diets. Second, we conducted long-read genome sequencing and analysis of the genome of the dominant, putative *Klebsiella* (hereafter ‘Medkleb’) spp. extracted from the adult gut of wild-type individuals. This was done to confirm the phylogenetic placement of Medkleb and to identify whether its genome showed any features characteristic of nascent evolutionary interdependence with its medfly host, such as a reduction in genome size or GC content [2–4]. Third, we conducted comparisons between Medkleb and other *Klebsiella* spp. to reveal phenotypes that might potentially facilitate a mutualistic relationship, or indicate restrictions to the environments in which Medkleb might live. Finally, we investigated phenotypic features of Medkleb by testing whether it had the capacity to synthesise secondary metabolites, and by conducting direct biochemical tests for pectinolytic activity.

## Methods

### Characterisation of the medfly gut microbiome using Amplicon sequencing

#### Generation of laboratory and wild-derived gut microbiome samples

(*i) Toliman wild-type strain*. The Toliman strain originated from Guatemala and has been reared in the laboratory since 1990. Our colony has been maintained in non-overlapping generations in a controlled environment room (humidity 50±5 %, temperature 25±0.5 °C) on a 12 : 12 light:dark cycle for over 30 generations [45]. Under this regime, larvae are raised on a sugar-yeast-maize medium (1 % agar, 7.4 % sugar, 6.7 % maize, 4.75 % yeast, 2.5 % Nipagin (10 % in ethanol), 0.2 % propionic acid) and adults are given *ad libitum* access to food (3 : 1 w/w sugar/ yeast hydrolysate) and water.

(*ii) Effect of the larval diet on the adult microbiome in the Toliman wild-type*. We generated samples of wild-type flies raised on different larval diets in the presence and absence of antibiotics (Table S1, available in the online version of this article) for subsequent gut dissection and 16S rRNA amplicon sequencing of the gut microbiome. A pool of eggs was collected from a single Toliman wild-type cage over a 24 h period and allocated at random to one of the four larval diet treatments. Approximately 500 Toliman eggs were then placed on 100 ml of each larval diet in a 1/3 pint glass bottle, with three biological replicates per diet treatment. Three diets provided varying carbohydrate levels and sources, while maintaining the same yeast (~protein) level: Sucrose High Protein (SHP) diet, Glucose diet, Starch diet. The fourth diet had a sucrose carbohydrate base but only 60 % of the yeast content: Sucrose Low Protein, SLP (Table S1). Propionic acid was used as the food preservative [46]. Antibiotic diets contained a final concentration of 100 µg ml^−1^ kanamycin, 200 µg ml^−1^ ampicillin, 200 µg ml^−1^ streptomycin, 50 µg ml^−1^ chloramphenicol, 100 µg ml^−1^ apramycin, 100 µg ml^−1^ hygromycin and 200 µg ml^−1^ tetracycline. Adults emerging from each replicate of each of the eight diet treatments were collected and maintained in groups of roughly 30 males and 30 females in plastic cages (11 cm × 11cm × 10 cm). Adults were fed a standard diet (*ad libitum* access to sucroseyeast food as above). Females (five per replicate) were sampled for gut dissection (below) from the cages at 3 days old (all eight diets) and 10 days old (four non-antibiotic diets only).

(*iii) Effect of wild larval diets on the adult microbiome in wild flies under natural conditions*. Wild flies of 0–3 days old were collected at adult eclosion from fallen argan fruit in Arzou, Ait Melloul, Morocco, in July 2014, from Apricots, Oranges and Grapefruits in Chania, Crete, July-September 2014 and from Peaches, Oranges and Tangerines in Ano Lechonia, Greece, July-September 2014. All samples were preserved in 96 % ethanol and sent to the UK before gut dissection of female flies (five females per each of the three replicates per fruit type) and DNA extraction for the 16S rRNA amplicon sequencing.

### 16s rRNA gene sequencing and bioinformatics analysis of the gut bacteria derived from laboratory- and wild-derived adult medflies

We analysed the composition of the gut microbiomes in the dissected guts of the laboratory- and wild-derived females described above, by using 16S rRNA amplicon sequencing. Gut dissections (extraction of the whole gut from crop to anus) were performed in sterile PBS with sterilised dissection tools. All dissection work was carried out inside a microbiological safety cabinet. Guts were surface sterilized for 30 s in 0.5 % sodium hypochlorite (bleach) (Sigma-Aldrich, Cat. No.7681529) and washed for 30 s in sterile 1M PBS (pH 7.4) three times before being homogenized. A 100 µl sample of the third washes were used to check the surface sterilisation efficiency. There was no microbial growth in any of these tests. We used sterilised pestles to homogenise the extracted samples inside 2 ml microcentrifuge tubes, using three freeze/thaw cycles in liquid nitrogen. DNA was extracted using the DNeasy Blood and Tissue Kit (Qiagen) and quality checked using a NanoDrop (Thermoscientific Nanodrop 8000 Spectrophotometer). Approximately 100 ng of DNA per sample was used as the template for PCR amplification with bacterial universal primers 515F (5′-GTG CCA GCM GCC GCG GTA A-3′) and 806R (5′-GGA CTA CHV GGG TWT CTA AT-3) against the 16S rRNA gene. Amplicon sequencing was performed using paired-end 250 bp V2 chemistry (Illumina MiSeq platform, Earlham Institute provider).

Demultiplexed sequences were obtained using mothur v38.2 [47], following their standard MiSeq operating procedures. Sequence variants were assigned to operational taxonomic units (OTUs) at a 97 % similarity threshold. Taxonomy assignment of OTUs was performed using the Silva database (release 132). The minimum library sizes per sample were ~17K following quality control. All statistical analyses of amplicon data were conducted in R v3.6.2 [48] using the phyloseq [49], vegan [50] and tidyverse [51] packages. Sequences were rarefied to normalise library sizes. Alpha diversity was estimated using the Shannon species diversity index, calculated with phyloseq. We compared these indices, with the categorical predictors of age, diet and origin, using linear regression. We visualised differences in bacterial community structure among samples (beta diversity) using non-metric multidimensional scaling (NMDS) plots of Bray-Curtis distance matrices with 10 000 permutations, followed by analysis of treatment groups in a PERMANOVA design. The function *betadisper* was used to check for the homogeneity of group dispersion values.

### Genome sequencing of the dominant gut microbiome *Klebsiella* spp. symbiont (Medkleb) from the Toliman wild-type

#### We investigated the identity of the recurrent *Klebsiella* spp. bacterial symbiont through isolation of culturable *Klebsiella* spp. colonies from the Toliman strain

*(i) Clonal isolation of Medkleb.* Clonal isolates of *Klebsiella* spp. obtained from individuals of the Toliman wild-type strain were made by making homogenised samples of surface sterilized adults that had been reared under the standard conditions described above. Homogenated samples were plated onto Simmon’s Citrate LB Agar (with bromothymol blue as a colour indicator) - recommended for the isolation of *Klebsiella oxytoca and K. pneumoniae* [52]. Culture plates were prepared from 15 biological replicate samples. Cultures were checked for morphological uniformity and their identity was confirmed by using PCR amplification with universal bacterial primers 28F and 806R. Thirteen of the 15 isolates had identical 16S rRNA gene sequences and were blast-matched to *Klebsiella* and our most abundant OTU from the 16S rRNA amplicon sequencing. Two isolates contained colonies which blast-matched to *Pantoea* spp. We chose a single *Klebsiella* spp. colony at random as the source for genomic sequencing, as described below.

*(ii) DNA preparation of Medkleb for genome sequencing.* The single clonal isolate selected for genome sequencing was streaked onto LB media (15 g l^−1^ Agar; 5 g l^−1^ NaCl; 5 g l^−1^ yeast extract; 1.5 g l^−1^ glucose; 10 g l^−1^ tryptone), incubated overnight at 25 °C, then transferred into a 1.5 ml microcentrifuge tube containing 1 ml of 10 % glycerol. The sample was vortexed for 30 secs, then centrifuged at 12 000 r.p.m. for 10 min. Glycerol was removed and the pelleted bacteria re-suspended in 2 ml of SET buffer (65 % v/v molecular grade H_2_O (ThermoFisher); 20 % v/v Tris (pH8); 5 % v/v 5M NaCl; 5 % v/v 10 % SDS; 5 % v/v 0.5M EDTA) before transfer to a 15 ml falcon tube. Then 20 µg of lysozyme (Sigma) and 0.4 µg achromopeptidase (Sigma) suspended in 40 µl of molecular grade water (ThermoFisher) and 0.02 µg of RNase (Fermentas) were added. The sample was mixed gently and incubated at 37 °C for 2 h. Subsequently, 240 µl of 10 % sodium dodecyl sulphate and 56 µl of proteinase K (20 mg ml^−1^) were added, before a second incubation at 56 °C for 2 h, with manual mixing every 30 mins. Following, 800 µl of 5M NaCl and 2 ml of chloroform were added and the sample was mixed by hand for 10 mins, before centrifugation at 4000 r.p.m. for 12 mins. The aqueous phase was then carefully transferred to a fresh tube. DNA was precipitated in 0.6 vol isopropanol, then transferred to a 1.5 ml microfuge tube by pipette. The DNA sample was washed once with 70 % ethanol. The ethanol was then removed, and 1 ml of 70 % ethanol added and the DNA sample left to incubate overnight at 4 °C. The ethanol was again removed and DNA re-suspended in 200 µl of molecular grade water (ThermoFisher).

*(iii) Single molecule real time (SMRT/PacBio) Medkleb genome sequencing.* DNA purity, concentration, and average fragment size were analysed using Nanodrop (ThermoFisher), Qubit v2.0 (Invitrogen) and Agilent Tapestation 4200 (Agilent) respectively. DNA was fragmented using a G-tube (Covaris), and SMRTbell library construction was carried out using a Template Prep Kit 1.0 (PacBio). The library was then size selected to >7 kb using the BluePippin system (Sage Science). Sequencing was carried out on a Pacific Biosciences RSII instrument, using two RSII SMRTcells v3 and P6-C4 chemistry (PacBio, Earlham Institute provider). Each cell was sequenced using a 240 min movie, using the Magbead OCPW v1 protocol (PacBio).

*(iv) Medkleb genome assembly.* The Medkleb genome was assembled according to the Hierarchical Genome-Assembly Process (HGAP.3) protocol [53] as follows. (1) Mapping – BLASR [54] was used to map reads >500 bp with a read quality >0.8 to seed reads >6000 bp. (2) Pre-assembly - the Directed Acyclic Graph Consensus (DAGCon) algorithm [55] was used to produce a consensus sequence based on BLASR mapping. DAGCon then trimmed the consensus, producing an error-corrected pre-assembled read. (3) *De novo* genome assembly - the overlap-layout-consensus assembler Celera Assembler v8.1 was used to process the pre-assembled read into a draft assembly. 4) Final consensus - the draft assembly was polished using the Quiver multiread consensus algorithm [53]. (5) The final consensus sequence was then manually trimmed to circularise the genome and place the stop codon (TGA) of the dnaA gene at the 5′ terminus.

*(v) Medkleb genome quality control.* An estimated Quiver quality value (QV) for the Medkleb final consensus genome was provided (Earlham Institute). Genome completeness was estimated with both benchmarking universal single copy orthologues (BUSCO) software 3.0.0 [56], and CheckM [57]. The *Enterobacteriales* order and *Enterobacteriaceae* family were used as reference datasets for BUSCO and CheckM analyses respectively. The BUSCO and CheckM scores for the Medkleb genome were then benchmarked against six conspecific RefSeq genomes. Genome contamination was estimated with CheckM, and mlplasmids [58] was used to classify contigs as either chromosomal or plasmid DNA. The *Klebsiella pneumoniae* support-vector machine (SVM) model was utilised for the analysis, with minimum posterior probability specified at 0.7 and minimum contig length at 1000nt.

*(vi) Annotation and genome mapping.* Coding sequences within the Medkleb chromosomal DNA and plasmids, were called with the Prodigal algorithm [59]. Gene calls were then annotated with Classic-RAST [60]. Ribosomal RNAs (rRNAs) and transfer RNAs (tRNAs) were called and annotated with Classic-RAST. Circular maps were created for the Medkleb chromosomal DNA and plasmids using DNAplotter [61]. The putative plasmid sequence data were interrogated for plasmid origins of replication, using DoriC [62].

### Searching for signatures of symbiotic mutualisms in the Medkleb genome

In order to test whether the Medkleb genome carried any overt signatures of nascent mutualism, we compared the size, composition and synteny of the Medkleb genome with that of other published *Klebsiella* sequences.

(*i) Genome size and GC content*. The genome size and GC content of thirteen *Klebsiella* strains were analysed using Artemis software [63]. These data were then compiled and compared manually.

(*ii) Local genomic rearrangement*. To predict recently acquired Medkleb sequences, the genome was aligned with three closely related bacteria. Genomes were manually re-ordered to place the *dnaA* stop codon at the 5′ terminus. Synteny was then predicted with progressive Mauve [64].

### Phylogenetic analyses of the Medkleb genome sequence

(*i) 16 s rRNA gene sequence analyses*. The Medkleb genome was searched for regions homologous to the 16S rRNA gene sequence of *K. oxytoca* strain ATCC 13182 (NR_118853.1) using BLASTn [65]. The region with greatest homology to NR_118853.1 (nts 341370–342821) was then parsed with RNAmmer 1.2 [66] which predicts ribosomal genes. Sixty-one 16S rRNA gene nucleotide sequences representing 60 *Klebsiella* strains*, Pseudomonas aeruginosa* strain JB2 and a putative Medkleb 16S sequence were used to create a phylogeny with the silva ACT web app [67]. Where possible, non-redundant sequences were extracted from the silva rRNA gene database [68]. All sequences were almost complete (>1400 bp) and met the standard operating procedure for phylogenetic inference (SOPPI) quality criteria set out by Peplies *et al*. [69]. The tree was computed with the FastTree maximum likelihood programme [70] using the GTR evolutionary model, gamma distribution and local bootstrapping parameters [71]. The resulting phylogeny was constructed with FigTree 1.4.3 [72].

(ii) *Average Nucleotide Identity (ANI) analyses*. A total of 35 RefSeq whole genome entries extracted from the NCBI database [73], representing four *Klebsiella* species, were used to calculate a hierarchical clustering based on Average Nucleotide Identity [74]. The ANI Calculator [75] was used to compute the hierarchy using the BIONJ algorithm [76]. The tree was constructed with FigTree 1.4.3 [72].

(*iii) Kleborate genotyping analysis*. Sixteen RefSeq genomes uploaded to the NCBI database [73] and designated as either *K oxytoca or K michiganensis* and the Medkleb genome, were parsed with Kleborate genotyping software [77].

### Metabolic functions of the medkleb *Klebsiella* gut symbiont

(*i) DNA preparation for PCR of pehX and 16S*. Medkleb and *Erwinia carotovora* bacteria were cultured in LB media (5 g l^−1^ NaCl; 5 g l^−1^ yeast extract; 1.5 g l^−1^ glucose; 10 g l^−1^ tryptone), *Rhizobium leguminosarum* was cultured in 2_X_TY media (16 g l^−1^ Tryptone; 10 g l^−1^ Yeast extract; 5 g l^−1^ NaCl). All media was stored in 50 ml glass bottles and autoclaved. Prior to use, each bottle was inoculated with a ‘loop’ of bacteria and incubated, shaking, in an orbital incubator (New Brunswick Scientific Innova 44) at 200 r.p.m. Medkleb and *Erwinia carotovora* were incubated at 37 °C and *Rhizobium leguminosarum* was incubated at 28 °C until optical density was greater than 1.0 at 600 nm.

(*ii) Analysis of presence of pehX and 16S rRNA genes in MedKleb*. DNA was extracted from 2 ml of liquid cultures of Medkleb, *Erwinia carotovora* and *Rhizobium leguminosarum* using a DNeasy blood and tissue kit (Qiagen) and microbe lysis buffer (MLB) (20 mg ml^−1^ of lysozyme (Sigma) and 5 mg ml^−1^ of achromopeptidase (Sigma) in 20 mM Tris-HCl, 2 mM EDTA, 1.2 % Triton X (pH 8.0)). The standard Qiagen protocol for animal tissue was followed but MLB used in place of ATL buffer and proteinase K. As in [78] the pehX gene was amplified using PEH-C forward primer (5′ GATACGGAGTATGCCTTTACGGTG 3′) and PEH-D reverse primer (5′ TAGCCTTTATCAAGCGG ATACTGG 3′). The 16S rRNA gene was amplified using 541F forward and 806R reverse primers, as in [79]. PCR parameters were the same for both assays: 20 µl PCR reactions were set up with 10 µl of Ultramix PCR buffer (PCR biosystems), 1 µl of forward primer (5 µM), 1 µl of reverse primer (5 µM), 1 µl of DNA (10 ng µl^−1^) and 5 µl of molecular water (ThermoFisher). Cycling parameters were: (1) 95 °C for 15 mins, (2) 35 cycles of 95 °C for 30 secs, 55 °C for 30 secs and 72 °C for 30 secs, (3) 72 °C for 10 mins. The *K. oxytoca* polygalacturonase gene pehX (AY065648.1) was aligned to genomes of *Klebsiella* bacteria using blast [65]. The presence of polygalacturonases in genomes of *Klebsiella* bacteria was assessed using the Carbohydrate-Active enZymes database (CAZy) [80].

(*iii) Polygalacturonase enzyme assay*. Medkleb’s ability to degrade pectin was compared to two bacterial species identified as positive and negative controls: *Erwinia carotovora* (+ve control) and *Rhizobium leguminosarum* (-ve control) [81, 82]. Polygalacturonase production of Medkleb, *Erwinia carotovora* and *Rhizobium leguminosarum* was measured using a DNS colorimetric method [83] with a protocol adapted from Sohail *et al*. [84] and Sigma Aldrich protocol EC 3.2.1.1. Cultures were diluted with LB broth to an optical density of 1.0 at 600 nm. Bacteria were then filtered from culture media with 0.2 µm PES syringe filters (ThermoFisher). Treatment reactions (which were run in triplicate) were set up with 1 ml of appropriate filtrate and 1 ml of polysaccharide solution (PS) (0.9 % polygalacturonic acid (ThermoFisher) in 0.1M sodium acetate (ThermoFisher) (pH 4.5)), a blank reaction was set up with 1 ml of PS only. All reactions were incubated at 45 °C for 30 mins before 1 ml of colour reagent solution (20 % 5.3M potassium sodium tartrate, tetrahydrate in 2M sodium hydroxide solution; 50 % 96 mM 3,5-Dinitrosalicylic acid solution; 30 % molecular water (ThermoFisher)) was added. All reactions were incubated at 100 °C for 15 mins, then placed on ice to cool to room temperature. Once cooled, 12 ml of molecular water (ThermoFisher) was added to each reaction, followed by hand mixing. Absorbance at 530 nm (ΔA530) was measured using a spectrophotometer (Biochrom) which had been blanked for air, with the corrected ΔA530 for treatment reactions being calculated as: *ΔA530 (Treatment reaction) = ΔA530 (Treatment reaction) – ΔA530 (Blank reaction)*.

Units of polygalacturonase in the filtrate were calculated via comparison to a standard curve of galacturonic acid (Sigma). Standards were made with between 50 µl and 1 ml of monosaccharide solution (MS) (1.8 % galacturonic acid (ThermoFisher) in 0.1M sodium acetate (ThermoFisher) (pH 4.5) and topped up to 2 ml total volume with molecular water (ThermoFisher). A standard blank was set up containing 2 ml of molecular biology grade water only. Then 1 ml of colour reagent solution was then added before incubation at 100 °C for 15 mins. Standards were placed on ice to cool to room temperature before 12 ml of molecular water was added and ΔA530 was measured using a spectrophotometer (Biochrom) which had been blanked for air. The corrected ΔA530 for standards was calculated as: *ΔA530 (Standard)=A530 (Standard) – A530 (Blank*). The standard curve was used to estimate milligrams of galactose released in treatment reactions with linear regression, and units of polygalacturonase per millilitre of filtrate were then calculated as: *Units/ml enzyme = (mg of galactose released)/ml of filtrate*).

(*iv) Prediction of higher order metabolic functions and secondary metabolites*. The higher order metabolic functions of genes were predicted using the Kyoto Encyclopaedia of Genes and Genomes (KEGG) [85]. Secondary metabolites were predicted using antiSMASH 6.0 beta [86].

## Results

### Characterisation of the medfly gut microbiome using Amplicon sequencing

**Fig. 1.**
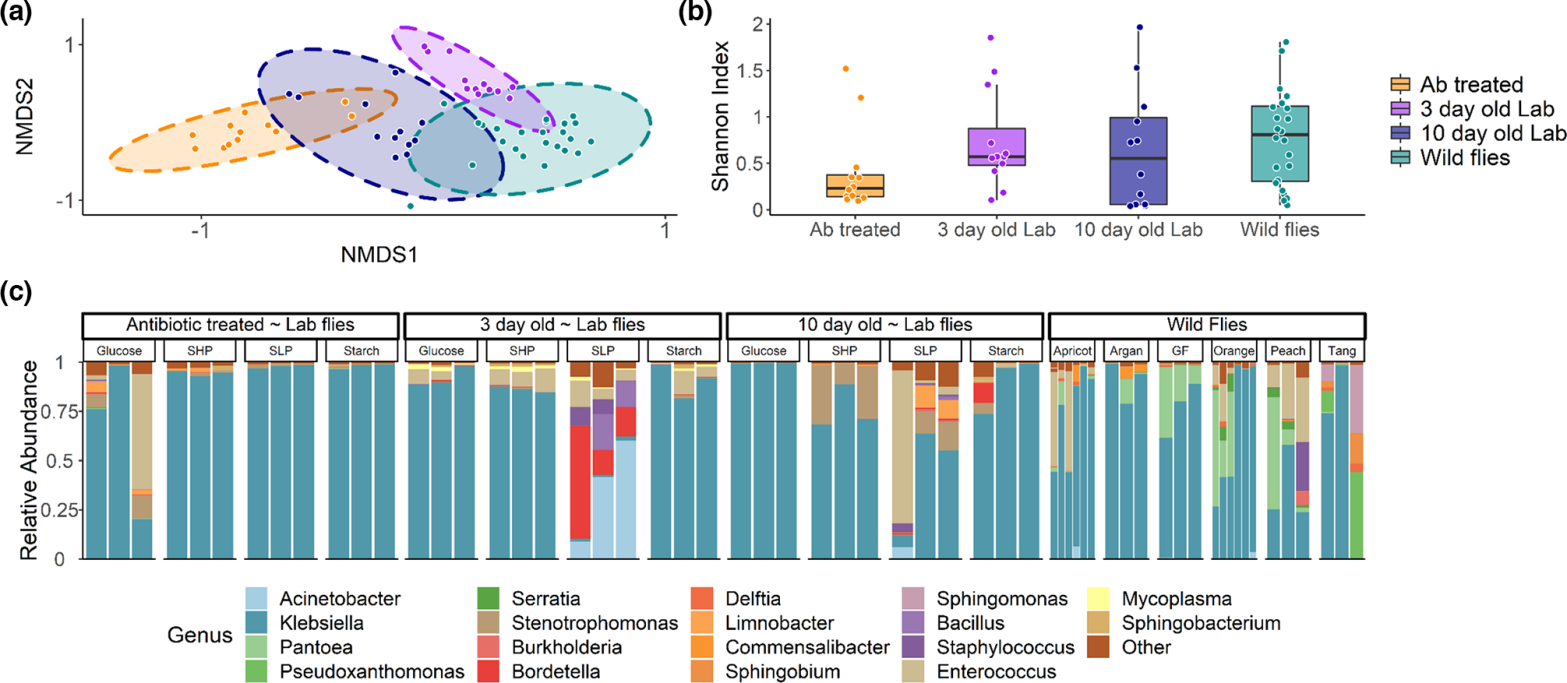
16S rRNA profiles of wild and laboratory strains of medfly on varied larval diets and in the presence and absence of antibiotic treatment. Microbiome composition was measured as (**a**) community structure/beta diversity visualised as NMDS plots using a Bray-Curtis Dissimilarity Index (stress value=0.19) with 95 % confidence ellipses (**b**) species richness/alpha diversity using the Shannon Index. Boxplot displays median, hinges are first and third quartiles, whiskers extend from hinge to 1.5× the interquartile range. (**c**) Bar plot of microbiome profiles. ‘Ab treated’=antibiotic treated 3 day old adult females from the Toliman laboratory strain (antibiotic treatment was 100 µg ml^−1^ kanamycin, 200 µg ml^−1^ ampicillin, 200 µg ml^−1^ streptomycin, 50 µg ml^−1^ chloramphenicol, 100 µg ml^−1^ apramycin, 100 µg ml^−1^ hygromycin and 200 µg ml^−1^ tetracycline); ‘3 day old Lab’=3 day old adult females from the Toliman laboratory strain; ‘10 day old Lab’=10 day old adult females from the Toliman laboratory strain. Laboratory larval diets are specified in Table S1. Three diets provided varying carbohydrate levels while maintaining the same yeast (~protein) level: (**i**) Sucrose High Protein (SHP), (ii) Glucose, (iii) Starch. The fourth diet comprised a sucrose carbohydrate with 60 % yeast: Sucrose Low Protein, SLP. ‘Wild flies’=adult females aged 0–3 days old collected from fallen argan fruit in Arzou, Ait Melloul, Morocco; Apricots, Oranges and Grapefruits in Chania, Crete; and from Peaches, Oranges and Tangerines in Ano Lechonia, Greece (*n*=5 females per replicate, three biological replicates.

(*i) Bacterial community diversity*. We found that OTU richness and diversity varied significantly among host samples according to treatment and origin (Fig. 1a, b). The full model included origin, diet, antibiotic treatment and age in sequential order and identified that community structure (beta diversity) was affected primarily by diet (PERMANOVA: F_8,50_ = 2.58, *P*=0.002, R^2^=0.27, Figs 1a and Fig. S1)and origin (F_1,48_ = 4.65, *P*=0.003, R^2^=0.06). Age and antibiotic treatment were manipulated only in the laboratory flies and a separate analysis to subset the data showed that age (F_1,30_ = 4.9, *P*<0.001, R^2^=0.18), diet (F_3,30_ = 2.03, *P*=0.023, R^2^=0.12) and antibiotic treatment (F_1,30_ = 4.9, *P*=0.002, R^2^=0.09) all affected community structure. The SLP diet in particular seemed to have a marked effect on microbiome profiles in 3 and 10 day old flies (Fig. 1c). This may suggest that the balance of carbohydrate to yeast in the larval diet may be key to determining microbiome composition, even more than the type of carbohydrate itself. In the analysis of the wild flies around half of OTU richness and diversity was accounted for by diet (F_5,16_ = 4.33, *P*<0.001, R^2^=0.51). Bacterial species diversity (alpha diversity) did not vary significantly in the wild or laboratory population strains but was lower in antibiotic treated flies. However, the Shannon index was not significantly different between any samples (Fig. 1b). Overall, we found no evidence of large-scale changes in diversity or composition of the microbiome despite testing multiple wild food sources, effects of laboratory diets and antibiotic treatment (Fig. 1c).

(*ii) Dominant bacterial taxa*. Four bacterial families representing two bacterial phyla made up over 90 % of the sequences in our dataset. These were the Proteobacteria, Enterobacteriaceae (79 %), Moraxellaceae (2.3 %) and Xanthomonadaceae (3.6 %), and the Firmicutes, Enterococcus (7.3 %) (Fig. 1c). Of these, a single bacterial genus of *Klebsiella* spp. emerged as a core member of the bacterial microbiome. A putative *Klebsiella* spp. was found in every medfly population sample and comprised 73.6 % of the entire dataset. This suggests that, although medflies are extremely polyphagous, they have a stable microbiome, containing a recurrent *Klebsiella* spp. symbiont. A limitation to our analyses was that an extraction blank was not performed for the 16S amplicon analysis to estimate the background microbial community derived from the method itself. However, we did run culture negatives, none of which generated any products when run on a gel, which suggests that the results are not strongly contaminated by background.

### Genome sequencing of the dominant gut microbiome *Klebsiella* spp. symbiont (Medkleb) from the Toliman wild-type

**Fig. 2.**
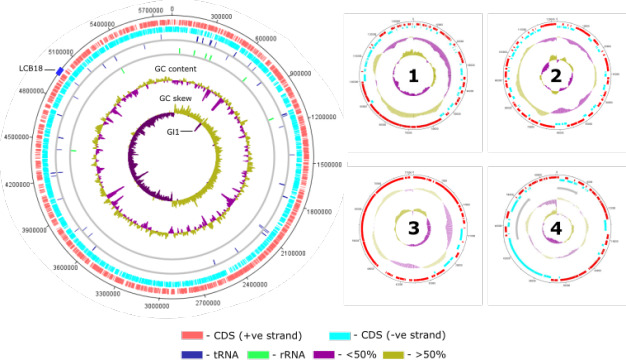
Circular summary map of the Medkleb chromosome and plasmids. For all maps: 1) red sectors on track one represent gene coding sequences on the positive strand, 2) light blue sectors on track two represent gene coding sequences on the negative strand, 3) GC content is represented by the penultimate track. Regions with >50 % GC content are coloured mustard and regions with <50 % GC content are purple, 4) the innermost track represents GC skew; regions with >50 % G’s are mustard and regions with <50 % G’s are purple. The Medkleb chromosome is represented with the stop codon (TGA) of the *dnaA* gene at position 0. Track one; 2541 red ticks represent gene coding sequences on the positive strand. Track two; 2847 light blue ticks represent gene coding sequences on the negative strand. Track three; 50 dark blue ticks above the grey line represent tRNAs on the positive strand, and 35 ticks below the line represent tRNAs on the negative strand. Track four; 18 green ticks above the grey line represent rRNAs on the positive strand, and seven ticks below the line represent rRNAs on the negative strand. A total of 64 % of rRNAs are found close to *oriC*. GC skew was asymmetric between leading and lagging strands, with the purple spike between bases 654265–677 695 (GI1) representing recent horizontal gene transfer [95]. The LCB18 gene island (≈nts 4.97×10^6^~5 x 10^6^) is represented by a blue block on the outer ring. Interesting individual plasmid features include: 1) mkp4 contains two secondary metabolite clusters indicated by grey arrows, 2) mkps 1, 3 and 4 exhibit clear coding bias.

(*i) Classification of Medkleb sequencing contigs*. Total Medkleb DNA was sequenced on a PacBio RSII module and assembled using the HGAP.3 algorithm and Quiver [53]. This process detected one large contig that sequestered 94 % of total gene space (Fig. 2) and four small contigs (mkp1-4; Fig. 2). This suggests that the total Medkleb DNA complement is formed of one chromosome and four plasmids. Consistent with this, the mlplasmids software [58] classified the large Medkleb contig as chromosomal and the four smaller contigs as plasmids (Table S2). In addition, mkp2, mkp3 and mkp4 were sequenced with relatively high coverage depth (Fig. S2) and showed evidence for high copy numbers, a common plasmid trait [87]. Finally, mkp3 and mkp5 were demonstrated to have low GC content relative to the putative chromosome (Table S2), which again is characteristic of plasmid DNA [88]. mkp2 and mkp4 both exhibited a similar GC content to that of the chromosome, suggesting that they have been acquired more recently [89].

(*ii) Quality control*. PacBio coverage depth >100X is considered sufficient for resolving nucleotide sequences [90] and this threshold was met by all contigs other than mkp4 (Fig. S1). The QV sequence resolution was 48.9 (an average error rate of one base in every 80100) and hence the Medkleb chromosome was of high quality (Fig. S2). The plasmid QV’s were: mkp1: 48, mkp2: 47.3, mkp3: 45.3 and mkp4: 44.8 (Fig. S2). Although the plasmid sequences had lower resolution, they were robust, with accuracy >99.994 % in all cases. The completeness of the Medkleb genome was measured with BUSCO [56], which was used to search the assembly for 440 marker genes associated with bacteria of the *Enterobacteriales* order. BUSCO estimated that the Medkleb chromosomal sequence was 99.1 % complete (436 of 440 genes complete and single copy), far exceeding general quality thresholds [91]. CheckM [57] was used as a second method to assess the Medkleb chromosome for completeness and contamination. By using as reference 1005 marker genes associated with the family *Enterobacteriaceae *, the CheckM software estimated that the Medkleb genome was 100 % complete and 0.212 % contaminated, again far exceeding standard quality thresholds [91]. The Medkleb genome’s BUSCO and CheckM scores were typical for those of RefSeq *K michiganensis* specimens.

(iii) *Medkleb genome*. The Medkleb genome was 5 825 435 nt in length, with 5388 putative coding sequences and a GC content of 56.03 % (Fig. 2, Table S3). At 87.8 %, overall coding sequence was within the expected range [92], and, as predicted by Reva *et al.* [93], genes were distributed symmetrically between the two DNA strands. There were 2541 putative coding sequences on the positive strand, which were predicted to code for 2473 proteins, 50 tRNAs and 18 rRNAs. On the negative strand there were 2847 putative coding sequences predicted to code for 2805 proteins, 35 tRNAs and seven rRNAs. KEGG [85] predicts that the Medkleb genome encodes for genes with 2842 distinct molecular functions. The genes for 16 (64 %) ribosomal RNAs (rRNA) clustered between nts 62435–70 701 near the origin of replication (oriC). Medkleb’s GC content was 57.28 % for protein coding genes, 53.83 % for rRNA and 58.93 % for tRNA. Consistent with Lobry (1996) [94] GC skew was asymmetric, with an overrepresentation of Gs on the leading strand and Cs on the lagging strand, indicating that the genome is largely stable with few recent recombination events. However, there is one obvious exception, in which GC skew was inverted (>50 % C’s) between bases 654265–677 695. This is indicative of a recent introgression that has resulted in the acquisition of a new gene island (designated GI1) [95, 96].

(iv) *Medkleb plasmids*. The lengths and GC contents of mkps 1–4 (Table S2) were all within expected range (i.e. for plasmids associated with *Klebsiella oxytoca *). mkps 1–4 are predicted by KEGG [85] to code for 48 functional orthologues and devote 14–22 % of gene space to plasmid associated genes and mobile element coding sequences. This is substantial in comparison to the main chromosome, which only allocated 0.02 % to such features. mkps 1, 3 and 4 exhibited asymmetric gene distribution between DNA strands (coding bias) (Table S4), which is common for plasmid genomes [93]. The coding bias of mkp3 was particularly clear, with ~90 % of the total gene complement found on the positive strand. mkp4 was the only plasmid predicted by antiSMASH 6.0 beta [86] to code for secondary metabolites, which included cloacin [97] and colicin bacteriocins [98]. Analysis of the plasmid sequence data using DoriC [62] showed that all four putative plasmids contained sequences characterised on the RefSeq database as plasmid origins of replication.

### Searching for symbiotic signatures in the Medkleb genome

**Fig. 3.**
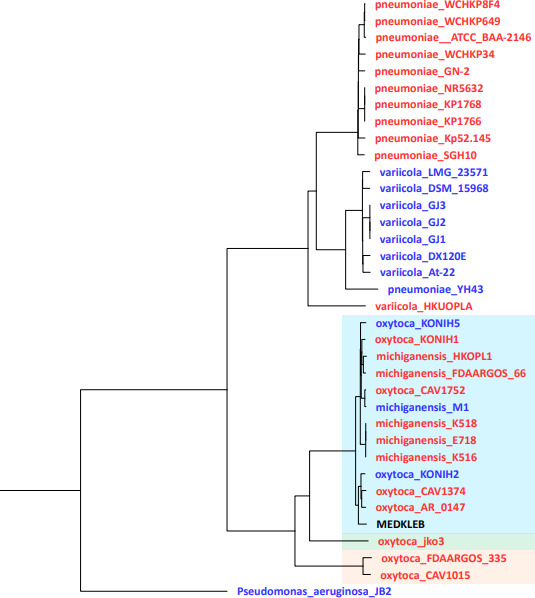
ANI hierarchical clustering showing the evolutionary relationship of environmentally-derived and host-derived *Klebsiella* bacteria. The tree was created using the ANI calculator [75], with *Pseudomonas aeruginosa* strain JB2 selected as the outgroup. Bacteria derived from animal hosts (red and black), and environmentally derived bacteria (blue), generally fell into three clades: 1) *K. pneumoniae *, 2) *K. variicola* and 3) *K. oxytoca/michiganensis*. With one exception in each group (YH43 and HKUOPLA), all *K. pneumoniae* are host derived and all *K. variicola* are environmentally derived. *K. oxytoca/K. michiganensis* have been isolated from both environmental and animal sources, but their sequences did not cluster according to source status. According to the ANI species threshold set by Kim *et al.* [105], Medkleb is conspecific with twelve strains in the ‘Medkleb group’ which have been classified on the RefSeq database as both *K. oxytoca* and *K. michiganensis *. However, *Kleborate* [77] identified all strains in the Medkleb group (bounded within a blue box) as *K. michiganensis,* the single strain bounded in a green box as *K. grimontii* and the two strains bounded in an orange box as *K. oxytoca *.

(i) *Genome size and GC content*. At 5 825 435 bp the Medkleb genome (Fig. 3) was the smallest in the ‘Medkleb group’ and 1.6 standard deviations smaller than the mean genome size of the clade (*n*=13) (x̄=6.07 x 10^6^ bp±1.49 x 10^5^ s.d.). However, we concluded this is not diagnostic of symbiotic transition as the genome of free-living *K. michiganensis* strain M1, was only 0.7 % larger than Medkleb (5.86×10^6^ bp). Medkleb’s GC content (56.03%) was also high, even for bacteria with a free-living life history [4] (Table 1). We conclude that there were no obvious diagnostic signatures of a strong evolutionary co-association.

**Table 1.** Genomic features of the ‘Medkleb group’ of bacteria. All genomes available on the CAZy database [80] are predicted to code for two pectate lyases. Medkleb had the smallest genome in the group^1^, and the second highest GC content^2^

NCBI species ID	Strain	Genome size (nt)	GC (%)	CAZy lyases
*oxytoca*	Medkleb	5825435*	56.03	na
*michiganensis*	M1	5 865 090	56.13†	2
*michiganensis*	HKOPL1	5 914 407	55.92	2
*oxytoca*	CAV1752	5 992 008	55.16	2
*michiganensis*	FDAARGOS_66	6 071 464	55.94	na
*michiganensis*	E718	6 097 032	56.02	2
*michiganensis*	K518	6 138 996	55.95	2
*michiganensis*	K516	6 139 574	55.96	2
*oxytoca*	KONIH1	6 152 190	55.91	2
*oxytoca*	KONIH5	6 179 177	55.81	2
*oxytoca*	KONIH2	6 190 364	55.77	2
*oxytoca*	CAV1374	6 257 473	55.75	2
*oxytoca*	AR_0147	6 350 620	55.57	2

*Smallest genome.

†Highest GC content.

(*ii) Local genomic rearrangement*. The Medkleb genome was aligned, using progressive Mauve [64] to three closely related strains (Fig. S3). This analysis revealed the presence of 21 local colinear blocks (LCBs) of conserved DNA in all four genomes (Fig. S3). These LCBs were not uniformly distributed between the genomes. Strains AR0147 and CAV1374 both contained inversions between LCBs 11–16 and strain KONIH2 contained several instances of translocation and inversion. However, despite inversion and translocation events, nucleotide sequence in all LCBs other than LCB18 was highly conserved. The annotation of Medkleb LCB18 (≈nts 4.97×10^6^~5×10^6^) predicts 25 coding sequences in total, including 13 ‘hypothetical proteins’, two DNA helicases (Mkleb_4576/4577), two methyltransferases (Mkleb_4579/4597) and an anti-restriction protein (Mkleb_4581). LCB18 may have been recently acquired as it also contains genes associated with horizontal transfer including an integrase (Mkleb_4574), a mobile element protein (Mkleb_4594) and a prophage protein (Mkleb_4585). In addition, two hypothetical proteins encoded by LCB18 (MKleb_4591 and Mkleb_4592) may be virulence factors, as they are predicted by TMHMM 2.0 [99] to encode N-terminal peptides. However, there is as yet no definitive evidence that LCB18 contains genes that might confer fitness to the medfly (Table 2). The Medkleb genome carries an inversion of GC skew on the leading strand (>50 % C; region GI1) indicating a putative horizontal gene transfer event. GI1 is thought to have been transferred from a plasmid, as it codes for *traY* which facilitates plasmid conjugal transfer [100]. Though plasmid integration into host chromosome is common [101, 102], GI1 does not appear to have integrated from any of the Medkleb plasmids (mkps 1–4) (Table 2). In total GI1 is predicted to code for 25 proteins including Bll0873, first sequenced in *Bradyrhizobium diazoefficiens *, a bacterium that is a known nitrogen fixing symbiont of legumes [103]. Interestingly, both GI1 (Mkleb_0586) and LCB18 (Mkleb_4596) code for genes predicted to facilitate molybdopterin biosynthesis, which could have the potential to benefit the medfly host, as this group of co-factors aid nitrate reduction [104]. According to KEGG analysis, the Medkleb genome contains coding sequences for 21 atypical functional orthologue genes that are not encoded in the genomes of any other closely related *Klebsiella* bacteria in the Medkleb group (Table 2 and Fig. S7). Some of these are potentially mutualistic functions such as sugar metabolism (*mtlA*) and nitrogen fixation (*nifZ*). Medkleb also has duplicates of 11 functional orthologues that *K. oxytoca* generally retains in only single copy. These duplicated functional orthologues do not cluster by genomic location. Several of these duplicated gene functions have strong mutualistic potential such as the biosynthesis of amino acids (*proC*, *trpA*) and breakdown of essential nutrients (*cite*, *pydC*). In contrast, Medkleb is also missing some clusters of genes that are present in closely related bacteria, e.g. for specific enzymes related to copper resistance (*copB, cusS, cusR, cusC, cusF*) and phosphonate transport (*phnC, phnD, phnE*).

**Table 2.** Unique features of the Medkleb genome in comparison to conspecifics

Unique feature	Identified via	Possible mutualistic function
*LCB18*	progressiveMauve analysis of Medkleb and three conspecifics	Codes for genes related to molybdopterin biosynthesis and several proteins with unknown function. These genes may confer fitness to the medfly.
*GI1*	Inversion of GC skew	Codes for genes related to molybdopterin biosynthesis and several proteins with unknown function. These genes may confer fitness to the medfly.
*Atypical metabolic genes*	Comparison of KEGG gene functions between Medkleb group members.	*mtlA*, *ACADSB*, *egsA* and *FAAH2* expand the range of nutrients available to the medfly.
*Absent sensory genes*	Comparison of KEGG gene functions between Medkleb group members.	The medfly gut may be relatively non-toxic allowing Medkleb to survive without genes that detect and metabolise certain chemical threats in its environment (*copB, cusS, cusR, cusC, cusF, arsB, nthA*).
*Duplicated genes*	Comparison of KEGG gene functions between Medkleb group members	Duplication of 11 genes associated with biosynthesis of amino acids and breakdown of essential nutrients. These genes may confer fitness to the medfly by providing access to extra nutritional resources
*Butyrolactone biosynthesis*	antiSMASH 6.0 beta	Possible regulation of antibiotic products [108, 109]
*N-acyl amino acid biosynthesis*	antiSMASH 6.0 beta	An important family of endogenous signalling molecules in which an amide bond covalently links an amino acid to the acyl moiety of a long-chain fatty acid. Primarily involved in cell-to-cell communication [110, 111]

### Phylogenetic analyses of the Medkleb *Klebsiella* gut symbiont genome sequence

(*i) Taxonomic identification of Medkleb –16S rRNA and ANI analysis*. Medkleb was predicted to be a strain of *K. oxytoca,* which has previously been proposed as a major component of the medfly microbiome [39]. The 16S rRNA gene sequence of *K. oxytoca* strain ATCC 13182 (NR_118853.1) was used to locate homologous sequences in the Medkleb genome using the BLASTn algorithm [65]. This revealed that the Medkleb genome contains eight sequences >99 % related to NR_118853.1, which were all predicted to code for 16S rRNAs by RNAmmer 1.2 [66]. Nucleotides 341370–342821 (mk16S) which had the greatest homology with NR_118853.1 were therefore used to represent Medkleb in a subsequent taxonomic analysis (as described in the Supplementary Information), which indicated that Medkleb was indeed likely to fall within a group of *Klebsiella oxytoca / michiganensis* spp. (Fig. S4).

A more stringent Average Nucleotide Identity (ANI) [74] analysis was also conducted. The Medkleb genome was positioned in an ANI matrix with 35 RefSeq *Klebsiella* genomes (Fig. S5 and Table S6) that were extracted from the NCBI database [73] (Fig. 3). This analysis placed Medkleb in a lineage with 13 strains classified as both *K. oxytoca* and *K. michiganensis *. ANI scores >95 % are required to classify bacteria as the same species [105, 106]. This 95 % similarity threshold was met by all thirteen members of Medkleb’s ANI clade (henceforth referred to as the Medkleb group) (Fig. 3). Hence both 16S rRNA gene and ANI analyses did not differentiate between *K. oxytoca* and *K. michiganensis* as they are currently named.

Finally, Kleborate [77] which assigns species to members of the *Klebsiella* complex via analysis of nine core loci and antimicrobial resistance markers, was used to analyse the Medkleb group and three closely related isolates. The Kleborate phylogenetic analysis replicated the ANI clustering precisely, placing Medkleb in a group of thirteen strains, which it identified as *K michiganensis *. Kleborate identified strain jko3 as *K. grimontii* and the FDAARGOS_335/CAV1015 node as *K. oxytoca *. Hence, ANI was effective at delineating species for all *Klebsiella* genomes analysed but Kleborate was required to resolve taxonomy.

### Metabolic functions of the Medkleb *Klebsiella* gut symbiont *K. michiganensis*

**Fig. 4.**
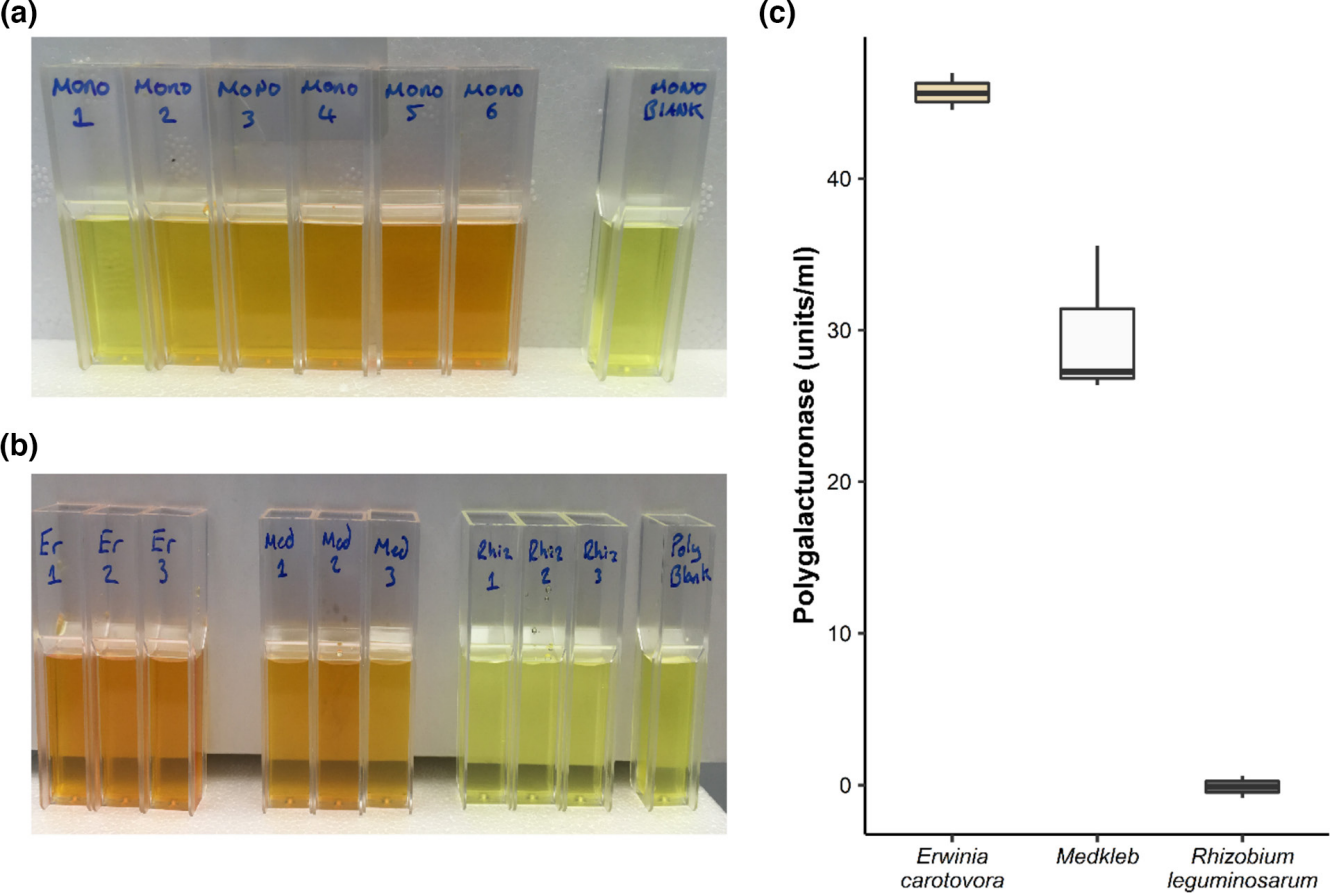
Polygalacturonase production by *Erwinia carotovora* (Er), Medkleb (Med) and *Rhizobium leguminosarum* (Rhiz). (a) Visual display of monosaccharide colorimetric reaction mixtures as a standard curve. The quantity of sugar in standards ranged from 0.9 mg (mono 1) to 18 mg (mono 6). Mono blank contained no sugar. When incubated with DNS, colour change (measured at ΔA530) occurred for all reactions relative to the blank. The relationship between sugar quantity and colour change was significant (linear model, F_1,4_ = 176.4, *P*<0.001, R^2^=0.98). (b) Visual display of bacterial reaction mixtures. Shown is the polygalacturonase production of three bacterial species: *Erwinia carotovora* (*Er*), Medkleb (*Med*) and *Rhizobium leguminosarum* (*Rhiz*) (three replicates of each) - measured via the quantity of reduced sugar in solution, following incubation of culture filtrate with polygalacturonic acid. Units of polygalacturonase were quantified in terms of colour change via extrapolation from the standard curve. *Med* and *Er* filtrate contained 30 and 46 units of polygalacturonase per millilitre respectively. *Rhiz* filtrate did not contain any polygalacturonase. (c) Plot of units of polygalacturonase contained in the bacterial filtrates. Units of polygalacturonase per millilitre are represented on the y-axis. The 1000 ul aliquots of bacterial filtrate are processed on the x-axis. Bacteria were assessed for the presence of polygalacturonases using a standard curve of galacturonic acid. The upper and lower hinges of boxplots represent the first and third quartiles of enzyme concentrations in filtrate, calculated from three technical replicates. *Er* was the highest producer, with an average of 45.7 units ml^−1^ and Med produced polygalacturonase with an average of 29.7 units ml^−1^. *Rhiz* did not produce polygalacturonase.

(*i) Polygalacturonase enzyme assay*. The ability to degrade pectin is suggested to be a defining phenotype of *K. oxytoca,* conferred by the polygalacturonase gene, pehX (AY065648.1) [78]. Conversely, *K. michiganensis* is reported to be pehX negative and unable to degrade pectin [107]. However, though our phylogenetic analyses identified Medkleb as *K. michiganensis *, it was also pehX positive (Fig. S6). To resolve this discrepancy, we conducted a functional test. We quantified Medkleb’s capacity to degrade polygalacturonic acid, in comparison to that of known positive (* E. carotovora *) and negative (* R. leguminosarum *) control species, using a DNS colorimetric method [83]. A standard curve of galacturonic acid incubated with DNS (Fig. 4a), showed a positive relationship with colour change when the OD was measured at 530 nm (linear model, F_1,4_ = 176.4, *P*<0.001, R^2^=0.97). Filtrate of Medkleb and *E. carotovora* culture media both contained pectinolytic enzymes, producing measurable colour change when incubated with polygalacturonic acid and DNS (Fig. 4b [84]). Medkleb filtrate reduced polygalacturonic acid with around 65 % the efficiency of the *Erwinia carotovora,* but *R. leguminosarum* filtrate did not demonstrate any measurable pectinolytic activity (Fig. 4c). These data suggest that at least some strains of *K. michiganensis* can degrade pectin, and that pectinolysis is not a reliable phenotype for discriminating between *Klebsiella* species.

(*ii) Predicted metabolic functions of Medkleb*. KEGG [85] was used to produce a list of gene functions for chromosomes and plasmids associated with all *K. oxytoca* bacteria in the Medkleb group (Table S5). The dataset was filtered to produce three lists: 1) Atypical - gene functions unique to Medkleb, 2) Absent - gene functions present in all genomes analysed other than Medkleb, 3) Duplicated – gene functions encoded in multiple copies by Medkleb but not by any other genome analysed. The Medkleb genome contained 21 atypical functional orthologues (Fig. S7), and of these, 16 were chromosomally derived and five located on plasmids. The atypical genes included two putative transposases, enzymes involved in various modes of metabolism (e.g. *mtlA, ACADSB, egsA, FAAH2*) and four transport protein genes (e.g. *natA* and *gatC*). When compared to conspecifics, Medkleb has 35 absent gene functions, seven of which may indicate adaptation to the relatively innoxious medfly gut (Fig. S7). The largest cluster of absent genes was associated with copper resistance (*copB, cusS, cusR, cusC, cusF*) and genes that degrade arsenic (*arsB*) and nitriles (*nthA*) were also missing. The Medkleb genome codes for multiple copies of 11 functional orthologues, that other *K. oxytoca* bacteria in the Medkleb group retain in single copy at most (Fig. S7). Medkleb’s duplicated genes may have the potential to promote mutualistic phenotypes for the medfly, as they syntheise amino acids (*proC, trpA*) and metabolise beneficial nutrients such as fatty acids (*ACAT*) and citrate (*citE*).

(*iii) Estimation of secondary metabolites*. The Medkleb genome and plasmid sequences were interrogated using antiSMASH 6.0 beta [86] for the presence of novel secondary metabolites. Chromosomes of all *K. oxytoca* members of the Medkleb group (other than KONIH2) coded for three common secondary metabolite clusters: 1) non-ribosomal polypeptide synthetase (30 % similarity to turnerbactin), 2) thiopeptide antibiotic (14 % similarity to O-antigen), 3) ribosomally synthesized and post-translationally modified peptides (RiPPs). The Medkleb chromosome contained two unique secondary metabolite clusters that were not present in any other analysed *K. oxytoca* chromosomes (i) a butyrolactone, a signalling molecule utilised by *Streptomyces* bacteria to regulate antibiotic production and cell cycle processes [108, 109], and (ii) an N-acyl amino acid cluster, common to soil dwelling bacteria and involved in cell-to-cell communication [110, 111] (Table 2). Plasmid mkp4 was also predicted to code for cloacin and colicin bacteriocins that were not coded by any other *K. oxytoca* strain in the Medkleb group. The cloacin cluster encoded on mkp4 contains two mobile elements (MKleb_5887, MKleb_5890) and is generally considered to be toxic for *Klebsiella* bacteria [97].

## Discussion

### The medfly microbiome

In this study, we used laboratory and field-reared adult medflies to characterise key features of the bacterial microbiome of this important agricultural pest. The 16S rRNA gene amplicon sequencing of culturable and non-culturable bacteria revealed that the overall species richness of the gut microbiome was fairly stable between laboratory-reared flies raised on different diets and wild flies. Direct comparisons of beta diversity indicated that larval diet, rather than exposure to antibiotics or wild vs laboratory rearing was the primary driver of microbial diversity in gut microbiomes. Wild flies obtained from different geographical regions and hosts, and flies reared on different substrates and exposed to antibiotic cocktails in the laboratory, contained largely the same bacterial families. The data suggest that although medflies are highly polyphagous, they have a stable microbiome that is dominated by the bacterial family *Enterobacteriaceae,* including a putative symbiont *Klebsiella* spp. These findings are consistent with previous analyses of medfly microbiomes made using culture-based methods [28, 39, 43, 112]. The picture may be more complex, however, as one recent next-generation sequencing study did not isolate *Klebsiella* in medfly microbiomes from wild populations [113] while another identified possible geographic or host-genetic structure links with bacterial dominance (with samples from Greece being dominated by *Klebsiella* spp.) [114]. We note that the 16S rRNA amplicon protocol assesses the diversity of the gut microbiome variation but does not give an absolute quantification of the level of gut microbes that are present. It is possible that diet manipulations and antibiotic treatments might alter the overall level of microbes in the gut, so future work should also assess this, potentially by testing the absolute level of reference strains within a sample. In addition, further work could usefully be done to probe the primary drivers of alpha diversity, potentially by additional comparisons of richness versus evenness across samples. This could give greater resolution into the reduced alpha diversity in antibiotic treated flies and effects on diversity of diet itself.

### Genome sequencing and analysis of the putative Medkleb gut symbiont

We obtained a fully sequenced Medkleb genome and both 16S rRNA gene and ANI analyses identified this as a *Klebsiella* species [105, 115]. Medkleb was *pehX* positive when analysed with PCR, and produced functional pectinolytic enzymes. Both of these characteristics suggest it is *K. oxytoca* [78, 107]. However, our ANI clustering and multi-locus species assignment analyses instead classify Medkleb as the close congener *K. michiganensis *.

Genome size and GC content are generally reduced when a bacterium adopts a facultative lifestyle [2]. We found that, although the Medkleb genome was the smallest of all *Klebsiella* bacteria in the ‘Medkleb group’, the free-living *K. michiganensis* strain M1 was only 40 kb (0.7%) larger. The Medkleb GC content was also the second highest in the ‘Medkleb group’, counter to what would be expected for a facultative mutualist. A strongly symbiotic transition is generally associated with an increased mutation rate, which causes facultative symbionts to occupy extended branches of phylogenetic trees [2]. There was no evidence for this here, as we observed that Medkleb was slightly, but not markedly, distinct from the three bacterial species most closely related to it.

We also used high resolution comparative genomic techniques to test for signals of putative mutualism. These analyses were designed to test: (i) whether Medkleb possessed genetic loci/functions that were absent from closely related *Klebsiella* bacteria and possessed an obvious mutualistic capacity for the fly, and (ii) if Medkleb was lacking genetic loci/functions found in all closely related free-living *Klebsiella* spp.*,* that would be considered necessary for life in varied environments. We also note, however, that gene loss may be unpredictable in the early stages of facultative mutualistic transitions [3].

The Medkleb genome contained two discrete regions (GI1 and LCB18) which may have been acquired comparatively recently, and are not typically found in *K. oxytoca / michiganensis*. Both GI1 and LCB18 contain genes associated with horizontal transfer and encode several unannotated hypothetical proteins with mutualistic potential. We found that Medkleb can degrade pectin, and that there are several atypical functions encoded exclusively in the Medkleb genome in comparison to conspecifics. These atypical genes are dispersed throughout the genome which suggests that they are ancestral and not recently acquired. Some of these genes are predicted to encode metabolic enzymes that could allow the medfly to utilise otherwise unattainable nutrients. We note, however, that though algorithms such as antiSMASH [86] can successfully predict secondary metabolite production systems, it is less powerful in estimating what the metabolites are and how they function. Hence additional experimental work is required to validate these predictions. Medkleb was also observed to contain several duplicated genes randomly distributed throughout the genome, which could provide the medfly with beneficial nutrients and amino acids. Together, these genomic signatures suggest that Medkleb is a symbiont that improves host fitness through increased access to vital nutrients. If substantiated, this could help to partially explain the success of the medfly as a generalist pest. In addition, it should be possible to use additional diagnostic tests to establish the carbon sources that can be used by Medkleb and test whether they are comparable with other strains, or whether they are more restrictive (which might itself indicate adaptation to the symbiotic lifestyle). However, potentially mutualistic traits are not evidence of mutualism itself and many signals that are commonly associated with facultative mutualism are not evident in the Medkleb genome. Therefore, there is insufficient evidence, so far, to definitively classify Medkleb as a facultative mutualist of the medfly.

*Klebsiella* bacteria appear to be found almost universally in the gut microbiomes of medflies [28, 116]. Therefore, Medkleb might provide an opportunity for paratransgenic control of the medfly. This study is one of only a handful of comprehensive characterisations of the bacterial symbionts of the tephritid fruit fly *C. capitata* using culture-independent methods, and the first to characterise the genome of a putative symbiont. Future studies should sample all developmental stages of the medfly, and sample more intensively to establish the potential for phylosymbiosis. Complementing this should be experimental studies to establish localisation of *Klebsiella* within the medfly gut, to determine transmission mechanisms and assess metabolic communication and host benefit [117].

## Supplementary Data

Supplementary material 1
