# Supplementary material for: Characterisation of the symbionts in the Mediterranean fruit fly gut
Source: Microb Genom. 2022 Apr 21;8(4):000801. doi: 10.1099/mgen.0.000801 (PMC9453069; doi:10.1099/mgen.0.000801)
Supplement: Supplementary material 1 [file mgen-8-0801-s001.pdf]

Characterisation of the symbionts in the Mediterranean fruit fly gut

Darrington, M.[1]*, Leftwich, P.T.[1]*, Holmes, N.A.[1,4], Friend, L.A.[1], Clarke N.V.E.[1], Worsley, S.F.[1], Margaritopolous, J.T.[2], Hogenhout, S.A.[3], Hutchings, M.I.[1,4] & Chapman, T^[1]

[1]School of Biological Sciences, University of East Anglia, Norwich Research Park, Norwich, NR4 7TJ, UK.

[2]Department of Plant Protection, Institute of Industrial and Fodder Crops, Hellenic Agricultural Organization–DEMETER, Volos, Greece

[3]Department of Crop Genetics, John Innes Centre, Norwich Research Park, NR4 7UH, Norwich, UK

[4]Department of Molecular Microbiology, John Innes Centre, Norwich Research Park, Norwich, NR4 7UH, UK.

*Joint first authors

ORCID: PTL 0000-0001-9500-6592

ORCID: TC 0000-0002-2401-8120

ORCID: NAH 0000-0002-4979-9680

ORCID: SFW 0000-0003-4736-0938

ORCID: SAH 0000-0003-1371-5606

ORCID: MH 0000-0001-6628-5940

ORCID: JM 0000-0002-5893-8400

^Correspondence: tracey.chapman@uea.ac.uk

Supporting Information

1. Taxonomic analysis of 16S rDNA sequences of medkleb

The EZBioCloud (Yoon et al., 2017) was searched for sequences homologous to Medkleb's putative 16S rDNA sequence from nucleotides 341370-342821 (mk16S). The 16S sequence with greatest homology to mk16S belonged to the W14 strain, which had been classified as *K. michiganensis*. Medkleb's predicted species classification was *K. oxytoca,* hence near identical 16S rRNA homology with *K. michiganensis* was unexpected*,* although it is recognised that 16S rRNA sequence similarity alone does not confirm species identification (Tindall et al., 2010). As recommended by Tindall et al. (2010), homology between mk16S and 16S rRNA sequences that had been classified as either *K. oxytoca* or *K. michiganensis* was calculated using EZBioCloud (Yoon et al., 2017) but this also failed to distinguish Medkleb as either species. mk16S was compared with 40 16S rRNA sequences classified as either *K. oxytoca* or *K. michiganensis* and was found to be between 98.5 and 99.93% related to all strains

analysed. Stackebrandt (2006) suggest that >98.7% similarity should be the threshold at which 16S rRNA sequences are considered to be conspecific but some authors have suggested thresholds need to be as high as 99.5% (Janda et al., 2007). In conclusion, Medkleb could not be specifically characterised as either *K. oxytoca* or *K. michiganensis* via pairwise analysis of its 16S rRNA sequence, as according to current taxonomic standards (Janda et al., 2007; Stackebrandt, 2006), the *K. oxytoca* and *K. michiganensis* strains analysed here were themselves conspecific.

To classify Medkleb's species with improved resolution, a comprehensive 16S rRNA phylogenetic analysis (Figure S4) was carried out. RefSeq 16S sequences used in the analysis had been classified as either *K. oxytoca, K. michiganensis* or *K. pneumoniae* (Quast et al., 2013), and a single strain of *Pseudomonas aeruginosa* was used as an ancestral root for the phylogeny. *K. pneumoniae* sequences were included as a control, as this species is closely related to *K. oxytoca* (Kovtunovych et al., 2003) but distant enough to be distinguished phylogenetically. Medkleb, *K. oxytoca* and *K. michiganensis* sequences formed one homogenous group and *K. pneumoniae* formed an outgroup, suggesting that Medkleb is both *K. oxytoca* and *K. michiganensis* and hence that the species dichotomy is artificial, as suggested above. However, even though 16S rRNA phylogenetic and pairwise analyses failed to delineate *K. oxytoca* from *K. michiganensis*, these species should be considered distinct, as DNA-DNA hybridisation between them is <70% (Saha et al., 2013).

References

Janda JM, Abbot SL (2007) 16S rRNA gene sequencing for bacterial identification in the diagnostic laboratory: pluses, perils, and pitfalls. J. Clinical Microbiol, 45:761-2764.

Kovtunovych G, Lytvynenko T, Negrutska V, et al (2003) Identification of *Klebsiella oxytoca* using a specific PCR assay targeting the polygalacturonase pehX gene. Res Microbiol 154:587–592. doi: 10.1016/S0923-2508(03)00148-7

Quast, C., Pruesse, E., Yilmaz, P., Gerken, J., Schweer, T., Yarza, P., . . . Glöckner, F. O. (2013). The silva ribosomal RNA gene database project: Improved data processing and web-based tools. Nucleic Acids Research, 41. doi:10.1093/nar/gks1219

Saha R, Farrance CE, Verghese B, et al (2013) *Klebsiella michiganensis* sp. nov., a new bacterium isolated from a tooth brush holder. Curr Microbiol 66:72–78. doi: 10.1007/s00284-012-0245-x

Stackebrandt E (2006) Taxonomic parameters revisited : tarnished gold standards. Microbiol Today 33:152–155.

Tindall, B. J., Rossello-Mora, R., Busse, H. J., Ludwig, W., & Kampfer, P. (2010). Notes on the characterization of prokaryote strains for taxonomic purposes. Int J Syst Evol Microbiol, 60(Pt 1), 249-266.doi:10.1099/ijs.0.016949-0

Yoon SH, Ha SM, Kwon S, Lim J, Kim Y, Se, H. Chun, J, (2017). Introducing EzBioCloud: a taxonomically united database of 16S rRNA gene sequences and whole-genome assemblies. Int J Sys Evol Microbiol, 67:1613.

Supplementary figures

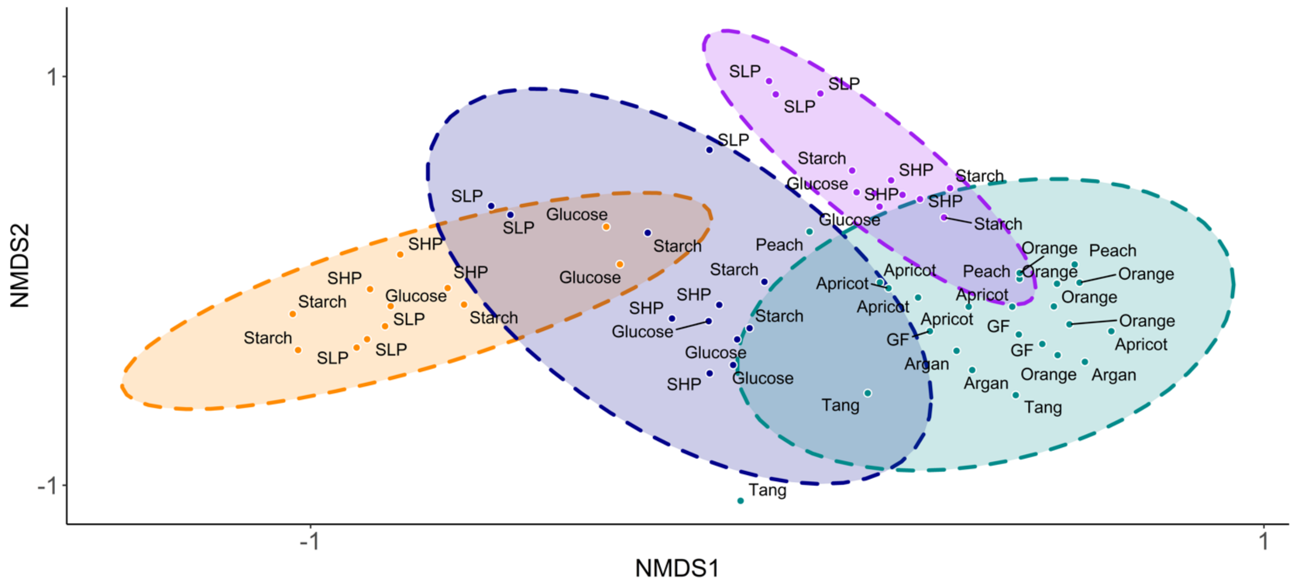

Figure S1. Medfly microbiome composition. Measured as community structure/beta diversity visualised as NMDS plots using a Bray-Curtis Dissimilarity Index (stress value = 0.19) with 95% confidence ellipses. The plot gives additional details of the data presented in the main Figure 1A, with the specific diet information added to each data point. Orange elipse = 3 day old laboratory antibiotic-treated females; mauve = 3 day old laboratory control (non-antibiotic treated) females; blue = 10 day old laboratory control (non-antibiotic treated) females; green = wild)-3 day old females.

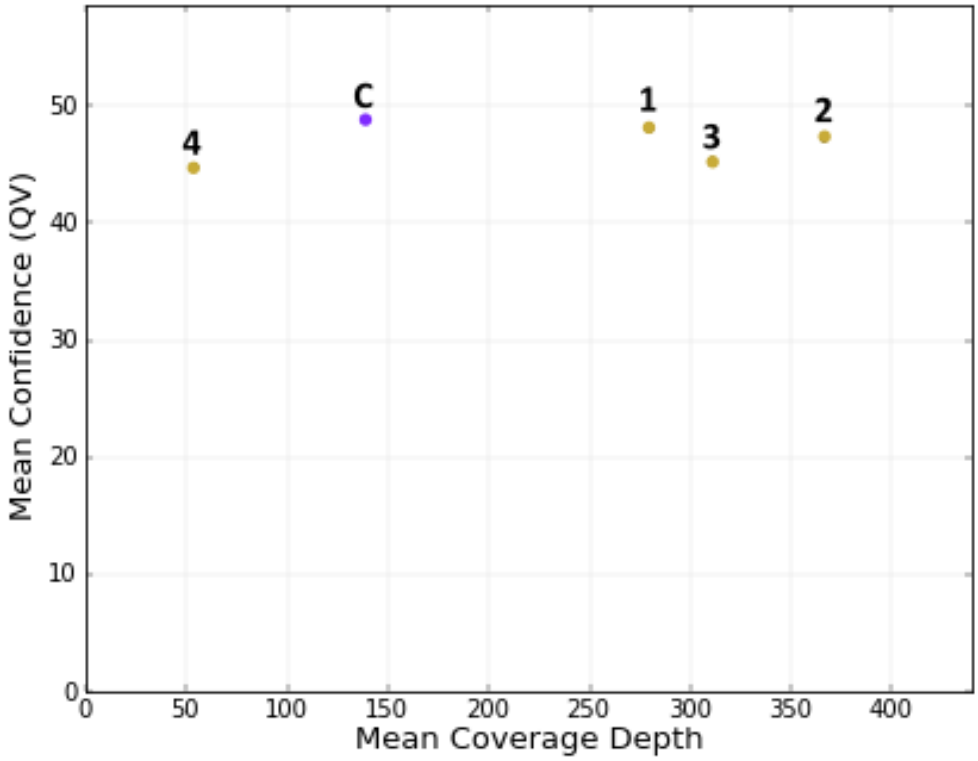

Figure S2. Contig coverage vs quality value for Medkleb sequencing contigs. The mean coverage depth for the Medkleb chromosome (purple dot) was 138.7 reads per base, and mean QV was estimated at 48.9. The four plasmids (yellow dots) had mean coverage depths and QV's of: mkp1) 279.6 and 48; mkp2) 366.4 and 47.3; mkp3) 310.8 and 45.3; mkp4) 53.6 and 44.8. Coverage was relatively high in the cases of mkp1-3, which was as predicted, as plasmids are often retained in high copy number.

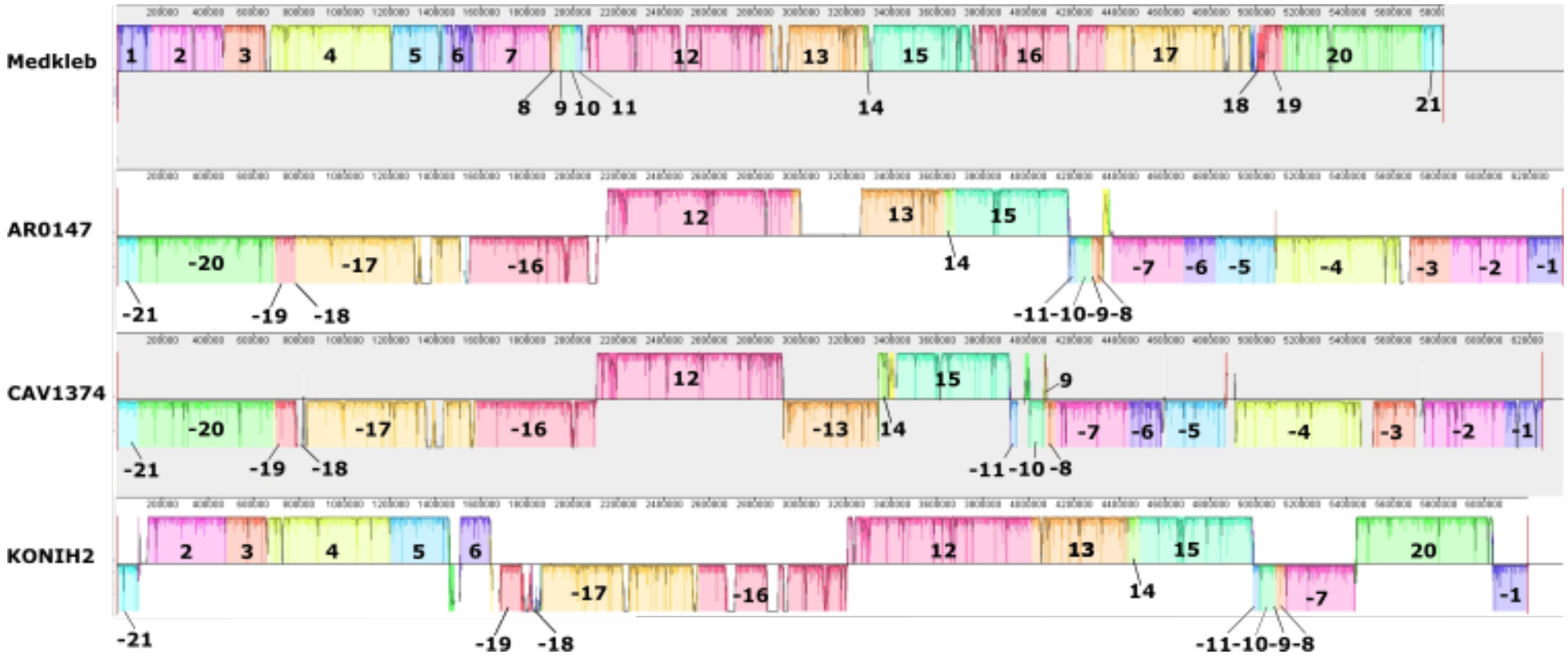

Figure S3 Synteny plot of Medkleb and three closely related bacteria. Medkleb was positioned phylogenetically in a clade with three *K. oxytoca* bacteria (strains AR0147, CAV1374 and KONIH2) with ANI analysis (Konstantinidis et al., 2005). progressiveMauve (Darling et al., 2010) was then used to assess synteny between Medkleb, AR0147, CAV1374 and KONIH2. This analysis, which is represented graphically above, uncovered 21 conserved local colinear blocks (LCBs) (Darling et al., 2010) of nucleotide sequence that were shared between all genomes analysed. Block height corresponds the degree of regional sequence conservation. When compared to Medkleb, sequence conservation in block 18 was low for strains AR0147, CAV1374 and KONIH2. There were several instances of LCB inversion between strains but inverted LCBs were not low in height, which indicated that nucleotide sequence was conserved.

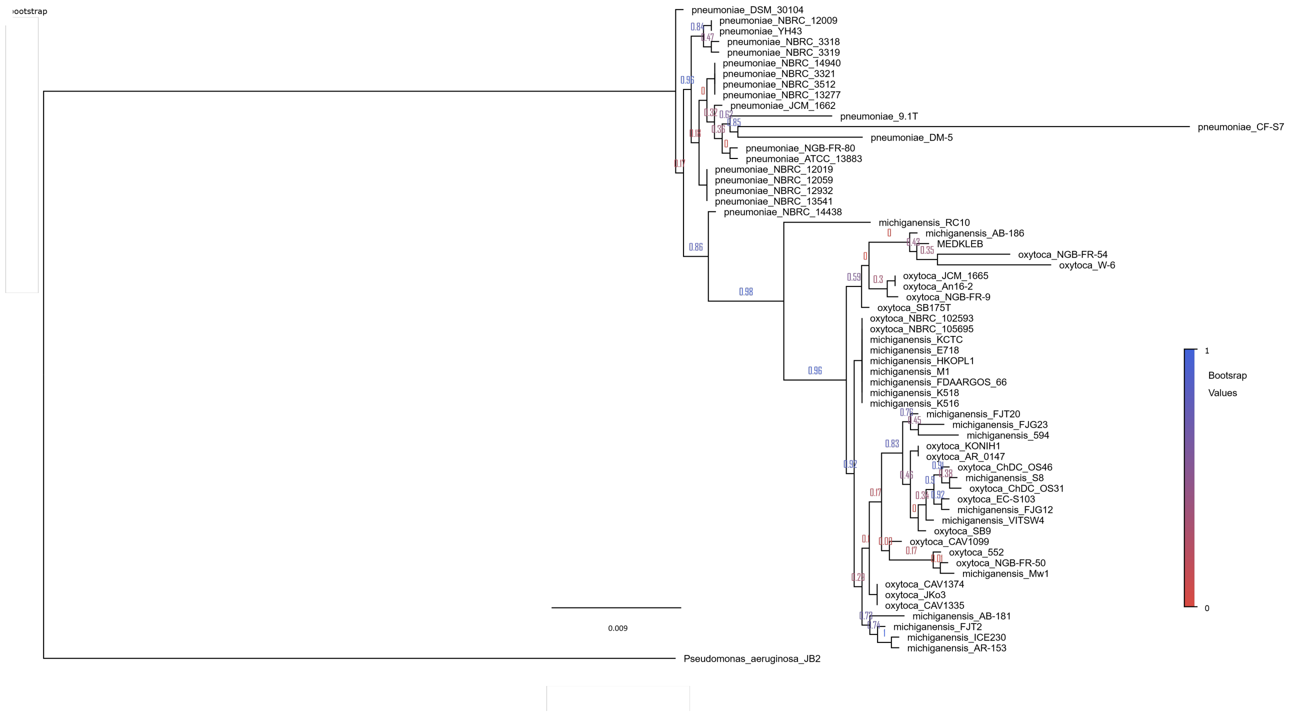

Figure S4. Phylogeny showing the evolutionary relationship of 16S rRNA genes belonging to Medkleb and three species of the *Klebsiella* genus. The phylogeny was created with the Silva ACT service (Pruesse et al., 2012), the FastTree2 maximum likelihood program (Price et al., 2010) and rooted with *Pseudomonas aeruginosa* strain JB2. The scale bar represents substitutions per site. Bootstrap values are represented at all nodes and are colour coded according to confidence level (blue = 1 and red = 0).16S rRNA sequences of strains that had been identified as *K. pneumoniae* demonstrated clear evolutionary divergence and segregated definitively from *K. oxytoca* and *K. michiganensis* strains. 16S rRNA sequences identified as *K. oxytoca* and *K. michiganensis* formed one homogenous group, with some species level clustering but no reliable pattern of distribution. Species identification of Medkleb was not possible based on this phylogeny, as it segregated within the *K. oxytoca, K. michiganensis* group.

	oxytoca_CAV1752	michiganensis_M1	oxytoca_KONIH5	oxytoca_KONIH1	michiganensis_FDAARGOS	michiganensis_HKOPL1	michiganensis_K518	michiganensis_K516	michiganensis_E718	oxytoca_CAV1374	oxytoca_AR_0147	oxytoca_KONIH2	MEDKLEB	oxytoca_jko3	oxytoca_FDAARGOS_335	oxytoca_CAV1015	Pseudomonas_aeruginosa_JB2	pneumoniae_SGH10	pneumoniae_Kp52.145	pneumoniae_WCHKP34	pneumoniae_GN-2	pneumoniae_KP1768	pneumoniae_KP1766	pneumoniae_NR5632	pneumoniae_WCHKP8F4	pneumoniae_WCHKP649	pneumoniae_ATCC BAA-2146	variicola_HKUOPLA	variicola_LMG 23571	variicola_At-22	variicola_DX120E	variicola_DSM 15968	variicola_GJ2	variicola_GJ1	variicola_GJ3	pneumoniae_YH43
oxytoca_CAV1752	100	100	99	100	99	99	99	99	99	99	99	99	99	93	92	92	76	84	84	84	84	84	84	84	84	84	84	84	84	84	84	84	84	84	84	84
michiganensis_M1	100	100	99	100	99	99	99	99	99	99	99	99	99	93	92	92	75	84	84	84	84	84	84	84	84	84	84	84	84	84	84	84	84	84	84	84
oxytoca_KONIH5	99	99	100	99	99	99	99	99	99	99	99	99	99	93	92	92	75	84	84	84	84	84	84	84	84	84	84	84	84	84	84	84	84	84	84	84
oxytoca_KONIH1	100	100	99	100	100	99	99	99	99	99	99	99	99	93	92	92	75	84	84	84	84	84	84	84	84	84	84	84	84	84	84	84	84	84	84	84
michiganensis_FDAARGOS	99	99	99	100	100	100	99	99	99	99	99	99	99	93	92	92	75	84	84	84	84	84	84	84	84	84	84	84	84	84	84	84	84	84	84	84
michiganensis_HKOPL1	99	99	99	99	100	100	99	99	99	99	99	99	99	93	92	92	75	84	84	84	84	84	84	84	84	84	84	84	84	84	84	84	84	84	84	84
michiganensis_K518	99	99	99	99	99	99	100	100	100	99	99	99	99	93	92	92	75	84	84	84	84	84	84	84	84	84	84	84	84	84	84	84	84	84	84	84
michiganensis_K516	99	99	99	99	99	99	100	100	100	99	99	99	99	93	92	92	75	84	84	84	84	84	84	84	84	84	84	84	84	84	84	84	84	84	84	84
michiganensis_E718	99	99	99	99	99	99	100	100	100	99	99	99	99	93	92	92	75	84	84	84	84	84	84	84	84	84	84	84	84	84	84	84	84	84	84	84
oxytoca_CAV1374	99	99	99	99	99	99	99	99	99	100	100	99	99	93	92	92	75	84	84	84	84	84	84	84	84	84	84	84	84	84	84	84	84	84	84	84
oxytoca_AR_0147	99	99	99	99	99	99	99	99	99	100	100	100	99	93	92	92	75	84	84	84	84	84	84	84	84	84	84	84	84	84	84	84	84	84	84	84
oxytoca_KONIH2	99	99	99	99	99	99	99	99	99	99	100	100	99	93	92	92	75	84	84	84	84	84	84	84	84	84	84	84	84	84	84	84	84	84	84	84
MEDKLEB	99	99	99	99	99	99	99	99	99	99	99	99	100	93	92	92	75	84	84	84	84	84	84	84	84	84	84	84	84	84	84	84	84	84	84	84
oxytoca_jko3	93	93	93	93	93	93	93	93	93	93	93	93	93	100	91	91	74	84	84	84	84	84	84	84	84	84	84	84	84	84	84	84	84	84	84	84
oxytoca_FDAARGOS_335	92	92	92	92	92	92	92	92	92	92	92	92	92	91	100	99	75	84	84	83	84	84	84	84	84	84	84	84	83	83	83	83	83	83	83	84
oxytoca_CAV1015	92	92	92	92	92	92	92	92	92	92	92	92	92	91	99	100	75	84	84	84	84	84	84	84	84	84	84	84	83	83	83	84	84	84	84	84
Pseudomonas_aeruginosa_JB2	76	75	75	75	75	75	75	75	75	75	75	75	75	74	75	75	100	75	75	75	75	75	75	75	75	75	75	75	75	75	75	75	75	75	75	75
pneumoniae_SGH10	84	84	84	84	84	84	84	84	84	84	84	84	84	84	84	84	75	100	99	99	99	99	99	99	99	99	99	93	94	94	94	94	94	94	94	94
pneumoniae_Kp52.145	84	84	84	84	84	84	84	84	84	84	84	84	84	84	84	84	75	99	100	99	99	99	99	99	99	99	99	93	94	94	94	94	94	94	94	94
pneumoniae_WCHKP34	84	84	84	84	84	84	84	84	84	84	84	84	84	84	83	84	75	99	99	100	99	99	99	99	99	99	99	93	94	94	94	94	94	94	94	94
pneumoniae_GN-2	84	84	84	84	84	84	84	84	84	84	84	84	84	84	84	84	75	99	99	99	100	99	99	99	99	99	99	93	94	94	94	94	94	94	94	94
pneumoniae_KP1768	84	84	84	84	84	84	84	84	84	84	84	84	84	84	84	84	75	99	99	99	99	100	100	100	99	99	99	93	94	94	94	94	94	94	94	94
pneumoniae_KP1766	84	84	84	84	84	84	84	84	84	84	84	84	84	84	84	84	75	99	99	99	99	100	100	100	99	99	99	93	94	94	94	94	94	94	94	94
pneumoniae_NR5632	84	84	84	84	84	84	84	84	84	84	84	84	84	84	84	84	75	99	99	99	99	100	100	100	99	99	99	93	94	94	94	94	94	94	94	94
pneumoniae_WCHKP8F4	84	84	84	84	84	84	84	84	84	84	84	84	84	84	84	84	75	99	99	99	99	99	99	99	100	100	100	93	94	94	94	94	94	94	94	94
pneumoniae_WCHKP649	84	84	84	84	84	84	84	84	84	84	84	84	84	84	84	84	75	99	99	99	99	99	99	99	100	100	100	93	94	94	94	94	94	94	94	94
pneumoniae_ ATCC BAA-2146	84	84	84	84	84	84	84	84	84	84	84	84	84	84	84	84	75	99	99	99	99	99	99	99	100	100	100	93	94	94	94	94	94	94	94	94
variicola_HKUOPLA	84	84	84	84	84	84	84	84	84	84	84	84	84	84	84	84	75	93	93	93	93	93	93	93	93	93	93	100	93	93	93	93	93	93	93	93
variicola_LMG 23571	84	84	84	84	84	84	84	84	84	84	84	84	84	84	83	83	75	94	94	94	94	94	94	94	94	94	94	93	100	99	99	99	99	99	99	97
variicola_At-22	84	84	84	84	84	84	84	84	84	84	84	84	84	84	83	83	75	94	94	94	94	94	94	94	94	94	94	93	99	100	99	99	99	99	99	97
variicola_DX120E	84	84	84	84	84	84	84	84	84	84	84	84	84	84	83	83	75	94	94	94	94	94	94	94	94	94	94	93	99	99	100	99	99	99	99	97
variicola_DSM 15968	84	84	84	84	84	84	84	84	84	84	84	84	84	84	83	84	75	94	94	94	94	94	94	94	94	94	94	93	99	99	99	100	99	99	99	97
variicola_GJ2	84	84	84	84	84	84	84	84	84	84	84	84	84	84	83	84	75	94	94	94	94	94	94	94	94	94	94	93	99	99	99	99	100	100	100	97
variicola_GJ1	84	84	84	84	84	84	84	84	84	84	84	84	84	84	83	84	75	94	94	94	94	94	94	94	94	94	94	93	99	99	99	99	100	100	100	97
variicola_GJ3	84	84	84	84	84	84	84	84	84	84	84	84	84	84	83	84	75	94	94	94	94	94	94	94	94	94	94	93	99	99	99	99	100	100	100	97
pneumoniae_YH43	84	84	84	84	84	84	84	84	84	84	84	84	84	84	84	84	75	94	94	94	94	94	94	94	94	94	94	93	97	97	97	97	97	97	97	100

Figure S5. Average nucleotide identity matrix for 35 strains of *Klebsiella* bacteria. The matrix was created using the ANI calculator (Figueras et al., 2014), with *Pseudomonas aeruginosa* strain JB2 selected as the outgroup. Not including the *Pseudomonas* outgroup, six distinct *Klebsiella* species were identified by the ANI analysis. Isolates with >95% ANI identity are considered conspecific and are highlighted pink. The “Medkleb group” is located at the top left of the matrix.

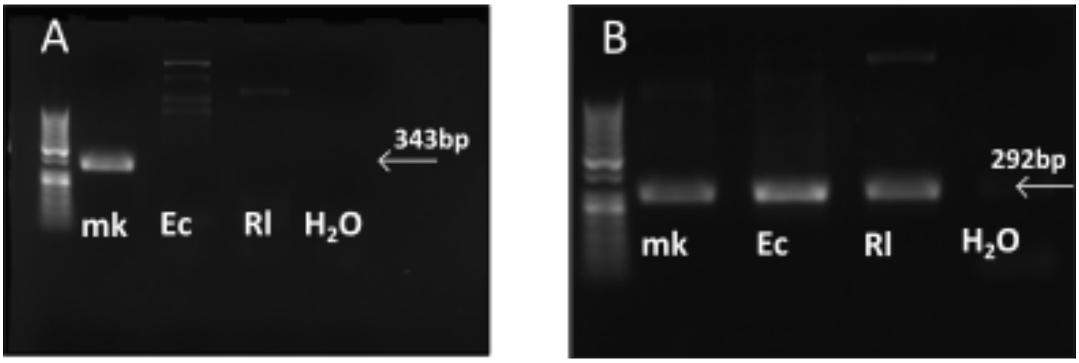

Figure S6. PCR amplifications of *pehX* and 16S rRNA genes. Both gels were run with three samples of DNA and a water control. A) PCR amplification of the *pehX* gene produced the expected 343bp product from Medkleb (*mk*). Neither *Erwinia carotovora* (*Ec*) nor *Rhizobium leguminosarum* (*Rl*) amplified this product, but both (*Ec* in particular) appeared to amplify products larger than 343bp. B) Control reactions: all three species of bacteria produced the expected 292bp 541F-806R amplicon following PCR amplification of 16S rRNA, which verified the quality of DNA and reagents used in the PCR assays. The assay was run mainly to help to identify Medkleb to species level, as the *pehX* primers deployed here have previously been used for that purpose (Kovtunovych et al 2003; Saha et al. 2013). Alternative primers could be designed for the *Erwinia pehX gene*. However, the purpose of the assay was as a diagnostic for Medkleb, not to explore any potential orthologues or alternative *pehX* gene possessed by *Erwinia* that might degrade pectin. For details of methods and primers, see main MS.

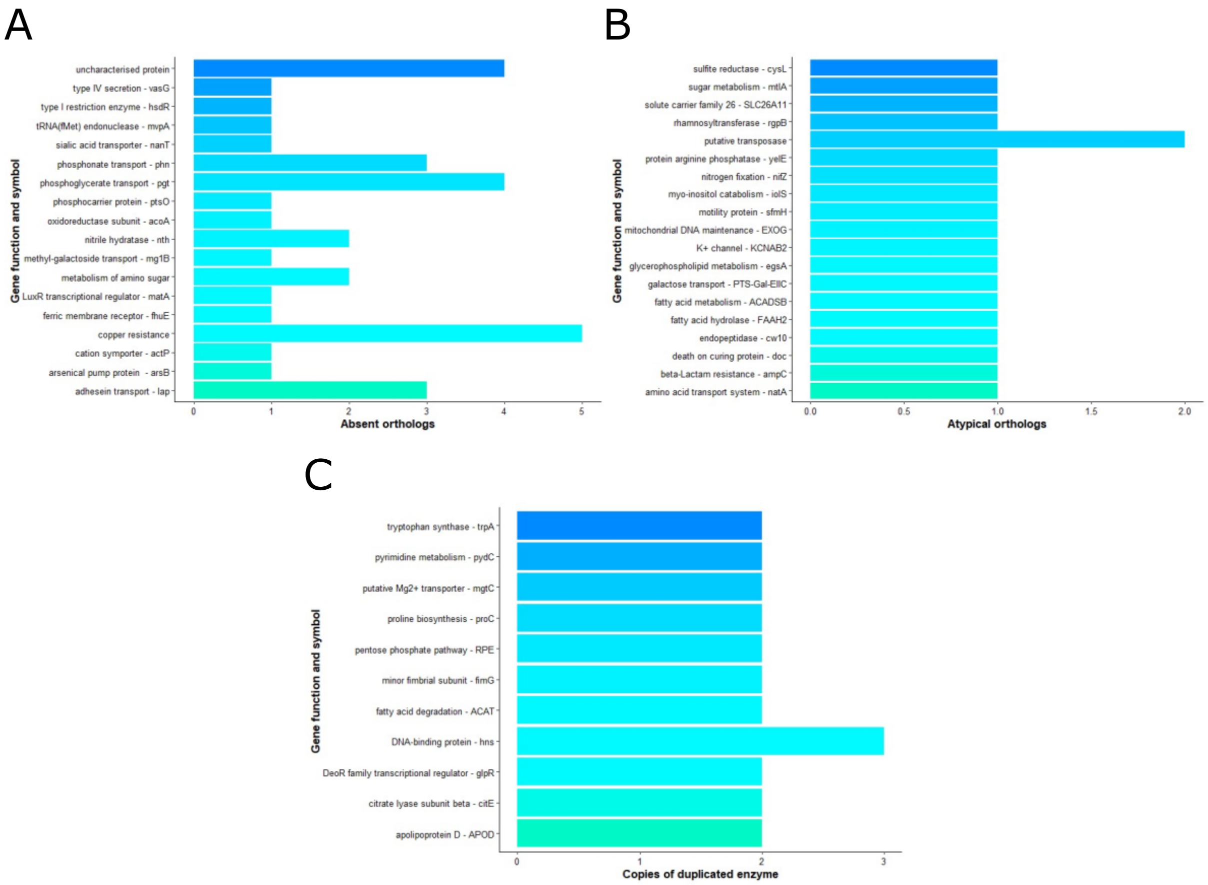

Figure S7. KEGG analyses of Medkleb's gene functions in comparison to six *K. michiganensis* specimens in the Medkleb group. The functions of genes and gene symbols are represented on the y-axes. Each function is denoted by a discrete colour. The number of genes providing each function are represented on the x-axes. **A)** Gene functions that were absent in the Medkleb genome but present in all other genomes analysed. Medkleb did not code for 35 genes that were present in every other genome analysed and these were associated with 17 discrete functions. Loss of function occurred in clusters of genes relating to the detoxification of xenobiotic compounds. **B)** Gene functions that were present in the Medkleb genome but absent from all other genomes analysed. Medkleb codes for 21 atypical genes with 20 different functions. Broadly, these genes were predicted to be involved in metabolism, transport and gene transposition. **C)** Gene functions that were duplicated in the Medkleb genome but not in any other genome analysed. Medkleb has duplicated 11 genes that are either absent or held in single copy by all other genomes analysed. Duplicated functions include the biosynthesis of amino acids and degradation of citrate.

Supplementary tables

Table S1. Larval diets. These larval diets were used for laboratory rearing of wild type Toliman flies for 16S rRNA investigation of adult female microbiomes**.**

Diet	Ingredient	Quantity
**Sucrose High protein (SHP)**	Water	1000 ml
	Agar	15 g
	Sucrose	30 g
	Yeast	50 g
	Propionic Acid	5 ml
**Sucrose Low Protein (SLP)**	Water	1000 ml
	Agar	15 g
	Sucrose	30 g
	Yeast	30 g
	Propionic Acid	5 g
**Starch**	Water	1000 ml
	Agar	15 g
	Starch	30 g
	Yeast	50 g
	Propionic Acid	5 g
**Glucose**	Water	1000 ml
	Agar	15 g
	Glucose	30 g
	Yeast	50 g
	Propionic Acid	5 g

Table S2. Contig derivation and GC content. mlplasmids (Arredondo-Alonso et al., 2018) predicted that the largest of the five Medkleb contigs was chromosomal, with a posterior probability (p(θ\X)) of 0.991. mkp2 and mkp5 were predicted to be plasmid-derived, with posterior probabilities of 0.962 and 0.977 respectively. The posterior probabilities of mkp3 and mkp4 being plasmid-derived were less powerful but still robust, at 0.864 and 0.882 respectively. mkp2 and mkp4 both had GC content comparable to that of the putative chromosomal contig, suggesting they had arisen as a result of a recent horizontal transfer.

**contig**	**length**	**p(θ\X)**	**Prediction**	**GC**(%)
1	5825435	0.009	Chromosome	56.03
2	136402	0.962	Plasmid (mkp1)	55.18
3	122124	0.864	Plasmid (mkp2)	50.4
4	78046	0.882	Plasmid (mkp3)	56.68
5	63257	0.977	Plasmid (mkp4)	50.65

Table S3. Synopsis of the Medkleb genome. The Medkleb genome was 5867451 nt in length and coded for a total of 5388 genes, at a coding density of 0.941 genes per kb.

**Length (nt)**	5825435
**GC content (%)**	56.03
**CDS (+ve strand)**	2541
**CDS (-ve strand)**	2847
**Overall coding sequence (%)**	87.8
**Gene density (per kb)**	0.941

Table S4. Comparison of plasmid genomes. mkps 1-4 varied in length by over 100%, and GC content varied by 6.3%. Plasmid 4 was relatively small but had the greatest gene density, utilising approximately 4% more nucleotide sequence for protein coding than other plasmids.

	**mkp1**	**mkp2**	**mkp3**	**mkp4**
**Length (nt)**	136402	122124	78046	63257
**GC content (%)**	55.2	50.4	56.7	50.7
**CDS (+ve strand)**	105	95	75	62
**CDS (-ve strand)**	70	64	8	42
**Overall coding sequence (%)**	80.3	81.1	81.2	85.3
**Gene density (per kb)**	1.28	1.31	1.06	1.64
**Annotated plasmid CDS (n)**	12	3	18	12
**Mobile element protein CDS [illegible]**	21	21	1	5

Table S5. Comparison of gene functionality for *K. oxytoca* bacteria in the Medkleb group. The total number of gene functions was fairly consistent in this group, at approximately 2900 per strain. In terms of atypical functions, Medkleb had a relatively large complement, but fewer than CAV1752. The Medkleb genome had more absent gene functions than was found for conspecifics.

	**Total gene functions**	**Atypical functions**	**Absent functions**
**Medkleb**	2857	21	35
**AR0147**	2904	12	4
**CAV1752**	2882	24	5
**CAV1374**	2944	11	6
**KONIH1**	2917	18	3
**KONIH2**	2912	13	10
**KONIH5**	2882	11	5

Table S6. Accession numbers for genomes and nucleotide sequences used for 16S and ANI analyses.

Strain	Classification on NCBI	Accession or nucleotides	Analysis
WCHKP8F4	pneumoniae	CP027068.2	ANI
WCHKP649	pneumoniae	CP026585.2	ANI
ATCC BAA-2146	pneumoniae	CP006659.2	ANI
WCHKP34	pneumoniae	CP025963.1	ANI
GN-2	pneumoniae	CP019160.1	ANI
NR5632	pneumoniae	CP025143.1	ANI
KP1768	pneumoniae	CP025140.1	ANI
KP1766	pneumoniae	CP025146.1	ANI
Kp52.145	pneumoniae	FO834906.1	ANI
SGH10	pneumoniae	CP025080.1	ANI
YH43	pneumoniae	NZ_AP014950.1	ANI
LMG 23571	variicola	CP013985.1	ANI
DSM 15968	variicola	CP010523.2	ANI
GJ3	variicola	CP017289.1	ANI
GJ2	variicola	CP017849.1	ANI
GJ1	variicola	CP017284.1	ANI
DX120E	variicola	CP009274.2	ANI
At-22	variicola	NC_013850.1	ANI
HKUOPLA	variicola	CP012252.1	ANI
KONIH5	oxytoca	CP026275.1	ANI
KONIH1	oxytoca	CP008788.1	ANI
HKOPL1	michiganensis	CP004887.1	ANI
FDAARGOS_66	michiganensis	JTBQ02000003.1	ANI
CAV1752	oxytoca	CP018362.1	ANI
M1	michiganensis	CP008841.1	ANI
K518	michiganensis	CP023185.1	ANI
E718	michiganensis	NC_018106.1	ANI
K516	michiganensis	CP022348.1	ANI
KONIH2	oxytoca	CP026285.1	ANI
CAV1374	oxytoca	CP011636.1	ANI
AR_0147	oxytoca	CP020358.1	ANI
JKo3	oxytoca	AP014951.1	ANI
FDAARGOS_335	oxytoca	CP027426.1	ANI
CAV1015	oxytoca	CP017928.1	ANI
CAV1374	oxytoca	NZ_CP011636.1	16S
jkO3	oxytoca	AP014951.1	16S
KONIH1	oxytoca	705389-706840	16S
AR0147	oxytoca	3961749-3963200	16S
JCM_1665	oxytoca	NR_113341.1	16S
NRBC_102593	oxytoca	NR_114152.1	16S
ECS103	oxytoca	AB200255.1	16S
An16-2	oxytoca	AB244452.1	16S
NBRC_105695	oxytoca	AB682268.1	16S
NGB-FR-9	oxytoca	AB749211.1	16S

NGB-FR-50	oxytoca	AB749216.1	16S
NGB-FR-54	oxytoca	AB749218.1	16S
W-6	oxytoca	AF390083.1	16S
ChDC_OS31	oxytoca	AF543283.1	16S
ChDC_OS46	oxytoca	AF543296.1	16S
SB9	oxytoca	AJ871855.1	16S
SB175T	oxytoca	AJ871858.1	16S
552	oxytoca	AY292867.1	16S
CAV1099	oxytoca	3582325-3583776	16S
CAV1335	oxytoca	3603336-3604787	16S
NGB-FR-80	pneumoniae	AB749220.1	16S
CF-S7	pneumoniae	AB933264.1	16S
DM-5	pneumoniae	AF390084.1	16S
DSM_30104	pneumoniae	AJ233420.1	16S
9.1T	pneumoniae	AY918488.1	16S
ATCC_13883	pneumoniae	NR_119278.1	16S
NBRC_14940	pneumoniae	NR_113702.1	16S
JCM_1662	pneumoniae	NR_113240.1	16S
NBRC_3318	pneumoniae	AB680060.1	16S
NBRC_3319	pneumoniae	AB680061.1	16S
NBRC_3321	pneumoniae	AB680063.1	16S
NBRC_3512	pneumoniae	AB680095.1	16S
NBRC_12009	pneumoniae	AB680212.1	16S
NBRC_14438	pneumoniae	AB680615	16S
NBRC_12019	pneumoniae	AB680217.1	16S
NBRC_12059	pneumoniae	AB680226.1	16S
NBRC_12932	pneumoniae	AB680340.1	16S
NBRC_13277	pneumoniae	AB680392.1	16S
NBRC_13541	pneumoniae	AB680431.1	16S
YH43	pneumoniae	5358317-5356853	16S
KCTC 1686	michiganensis	1556602-1558066	16S
E718	michiganensis	130024-131488	16S
HKOPL1	michiganensis	5479796-5481260	16S
M1	michiganensis	565404-566868	16S
RC10	michiganensis	4647101-4648565	16S
FDAARGOS_66	michiganensis	2604570-2606034	16S
K518	michiganensis	3135117-3136581	16S
K516	michiganensis	3014193-3015657	16S
VITSW4	michiganensis	KP100328.1	16S
S8	michiganensis	KX346260.1	16S
Mw1	michiganensis	LC191534.1	16S
ICE230	michiganensis	KX588579.1	16S
AB-186	michiganensis	KF817789.1	16S
AR-153	michiganensis	KF817762.1	16S
FJT2	michiganensis	MG571664.1	16S
AB-181	michiganensis	KF817784.1	16S
FJT20	michiganensis	MG571682.1	16S
FJG12	michiganensis	MG516124.1	16S

FJG23	michiganensis	MG516135.1	16S
594	michiganensis	KY407753.1	16S
